# Multimodal neuroimaging elucidates the posterior-to-anterior gradient of nigrostriatal degeneration in Parkinson’s disease

**DOI:** 10.1038/s41531-026-01456-y

**Published:** 2026-07-01

**Authors:** Huamei Lin, Ying Zhang, Yixin Zhao, Yiman Wei, Benjamin F. Combes, Martin T. Henrich, Fanni F. Geibl, Wolfgang H. Oertel, Kuangyu Shi, Axel Rominger, Ping Wu, Jingjie Ge, Jing Wang, Jiaying Lu, Zizhao Ju, Qian Xu, Huiwei Zhang, Yunhao Yang, Zengping Lin, Yang Yang, Yihui Guan, Fengtao Liu, Jian Wang, Jiehui Jiang, Ruiqing Ni, Chuantao Zuo

**Affiliations:** 1 Department of Nuclear Medicine/PET Center, Huashan Hospital, Fudan University, Shanghai, China; 2 School of Medicine, Shanghai University, Shanghai, China; 3 Institute of Biomedical Engineering, School of Life Sciences, Shanghai University, Shanghai, China; 4 School of Environmental and Chemical Engineering, Shanghai University, Shanghai, China; 5 Department of Neurology, Huashan Hospital, Fudan University, Shanghai, China; 6 United Imaging Healthcare Co., Ltd., Chengbei Road, Shanghai, China; 7 Institute for Biomedical Engineering, University, and ETH Zurich, Institute for Regenerative of Medicine, University of Zurich, Zurich, Switzerland; 8 Department of Psychiatry and Psychotherapy, Marburg University, Marburg, Germany; 9 Department of Neurology, Marburg University, Marburg, Germany; 10 Department of Nuclear Medicine, Inselspital, University of Bern, Bern, Switzerland; 11 Beijing United Imaging Healthcare Co., Ltd., Beijing, China

**Keywords:** Diseases, Neurology, Neuroscience

## Abstract

Parkinson’s disease (PD) exhibits a characteristic posterior-to-anterior gradient of striatal dopamine loss, yet the underlying upstream mechanisms remain unclear. We combined in vivo ¹⁸F-FPCIT dopamine transporter (DaT) PET/MRI, quantitative susceptibility mapping, and diffusion MRI tractography in 48 PD patients and 10 healthy controls to test whether substantia nigra pars compacta (SNc) iron and microstructural disruption mediate posterior putaminal vulnerability. PD patients showed marked posterior putamen (pPut) DaT reduction (~60%) and elevated SNc iron. SNc iron negatively correlated with striatal DaT availability, and nigrostriatal pathways to the pPut exhibited higher axial diffusivity. Serial mediation analyses demonstrated that SNc iron indirectly impaired striatal DaT function via reduced SNc DaT binding. Putaminal DaT-SBR robustly discriminated PD from controls (AUC 0.93–0.96), exceeding caudate performance. Transgenic and α-synuclein preformed fibril mouse models provided qualitative anatomical support. These findings identify SNc iron–related degeneration as a key driver of posterior striatal vulnerability in PD.

## Introduction

Parkinson’s disease (PD) is defined by progressive degeneration of substantia nigra pars compacta (SNc) dopaminergic neurons^1^, resulting in striatal dopamine depletion and the cardinal motor features of bradykinesia, rigidity, and tremor. Beyond terminal loss measurable with dopamine transporter (DaT) positron emission tomography (PET) (e.g., ¹⁸F-FPCIT), converging evidence indicates that SNc pathology—including iron accumulation and microstructural disorganization—may contribute to dopaminergic denervation^2^. Clinically and radiographically, a robust posterior-to-anterior gradient of striatal involvement has been consistently reported, with dopaminergic dysfunction first emerging in the posterior putamen—defined as the posterolateral subdivision of the putamen—and relative preservation of the anterior putamen in early PD^3^. This spatial pattern tracks motor symptom progression and implies selective vulnerability of nigrostriatal projections terminating in the putamen^4^. However, the mechanistic basis for this gradient—and the extent to which upstream SNc pathology drives region-specific striatal dysfunction—remains unresolved.

Iron deposition within the SNc—quantified with quantitative susceptibility mapping (QSM)—is a neuropathological hallmark of PD and has been implicated in processes related to oxidative stress^5^, and α-synuclein aggregation^6^. Diffusion MRI studies have demonstrated alterations in nigral tissue and nigrostriatal pathways in PD, reflected by changes in tensor-derived metrics such as axial diffusivity (AD), radial diffusivity (RD), mean diffusivity (MD), and fractional anisotropy (FA)^7^. Importantly, FA shows substantial between-study heterogeneity in PD^8^, and AD and RD are mathematically and biologically interdependent^9^, such that changes in one metric may influence the other, particularly in regions with complex fiber architecture. These SNc-centric abnormalities are hypothesized to propagate along the nigrostriatal pathway, ultimately manifesting as regional DaT loss in the striatum^10^. Yet, their subregional coupling to the posterior-dominant pattern of putaminal dysfunction remains insufficiently characterized.

Prior investigations typically examined SNc iron or diffusivity metrics in isolation, limiting mechanistic inference regarding their combined relationships to striatal DaT deficits and tract integrity^11^. Multimodal integration capable of testing a directed chain from SNc iron burden to nigrostriatal microstructural disruption and, in turn, to regional dopaminergic dysfunction has been scarce, and cross-species corroboration has been inconsistent. Moreover, few studies have explicitly modeled lateralized SNc–striatal relationships or posterior vs anterior putaminal susceptibility with harmonized regions of interest (ROIs) and cross-modal registration. These gaps preclude a mechanistically coherent account of the posterior putaminal vulnerability in early PD.

Here, we test a prespecified mechanistic hypothesis that SNc iron burden (QSM) is associated with dopaminergic integrity and microstructural properties within the nigrostriatal system, which together relate to the posterior-dominant pattern of striatal dopaminergic dysfunction (DaT PET) observed in PD. This framework is intended to examine cross-modal pathophysiological coupling within the nigrostriatal axis rather than to infer a strict temporal sequence of pathological events. In 48 PD and 10 healthy controls, we (i) quantify subregional DaT abnormalities emphasizing posterior vs anterior putamen and hemispheric effects; (ii) evaluate associations between SNc iron, SNc/tract microstructure, and striatal DaT; (iii) perform serial mediation analyses (indirect effects with bootstrap 95% CI; covariates including age/sex) to probe the directed chain; (iv) assess diagnostic discrimination using receiver operating characteristic (ROC)/area under the curve (AUC) with comparative testing for regional metrics; and (v) provide post-mortem and cross-species validation of SNc iron pathology using human tissue and transgenic as well as α-synuclein preformed fibril (PFF) mouse models with histological and MRI-based readouts. We anticipate that putaminal measures will outperform caudate metrics, and that SNc iron–iron microstructure coupling will account for a significant proportion of regional DaT variance.

## Results

### Participants

We enrolled 58 participants (PD, *n* = 48; healthy controls (HCs), *n* = 10). The PD cohort comprised 31 males/17 females, with an average age of 64.23 ± 9.31 years (range, 50–81 years), a disease duration of 4.53 ± 4.55 years, a UPDRS-III score of 37.52 ± 16.14, an MMSE score of 25.98 ± 3.52, and a levodopa equivalent daily dose (LEDD) of 391.09 ± 252.11 mg. Symptom lateralization was left-predominant in 16, right-predominant in 20, and non-lateralized in 12. HCs included 3 males/7 females, age 60.0 ± 7.05 years (47–78). No between-group differences were detected for age or sex (Table 1). Unless otherwise noted, all subsequent analyses used the full sample; per-analysis *n* and any exclusions are indicated in the figure/table legends.

**Table 1:** Demographic and clinical characteristics of study participants

	PD group (*n* = 48)	HC group (*n* = 10)	*p-*value	Cohen’s *d* (*d*)
Male/Female	31/17	3/7	0.075	0.716
Age (years)	64.23 ± 9.31	60 ± 7.05	0.883	-0.344
Disease duration (years)	4.53 ± 4.55	N/A	N/A	N/A
UPDRS-III score	37.52 ± 16.14	N/A	N/A	N/A
MMSE score	25.98 ± 3.52	N/A	N/A	N/A
LEDD (mg)	391.09 ± 252.11	N/A	N/A	N/A

*MMSE* mini-mental state examination, *HC* healthy control, *LEDD* levodopa equivalent daily dose, *PD* Parkinson’s disease, *UPDRS-III* Unified Parkinson’s Disease Rating Scale Part III.

### Primary striatal dopaminergic deficits (DaT-PET)

**Fig. 1:**
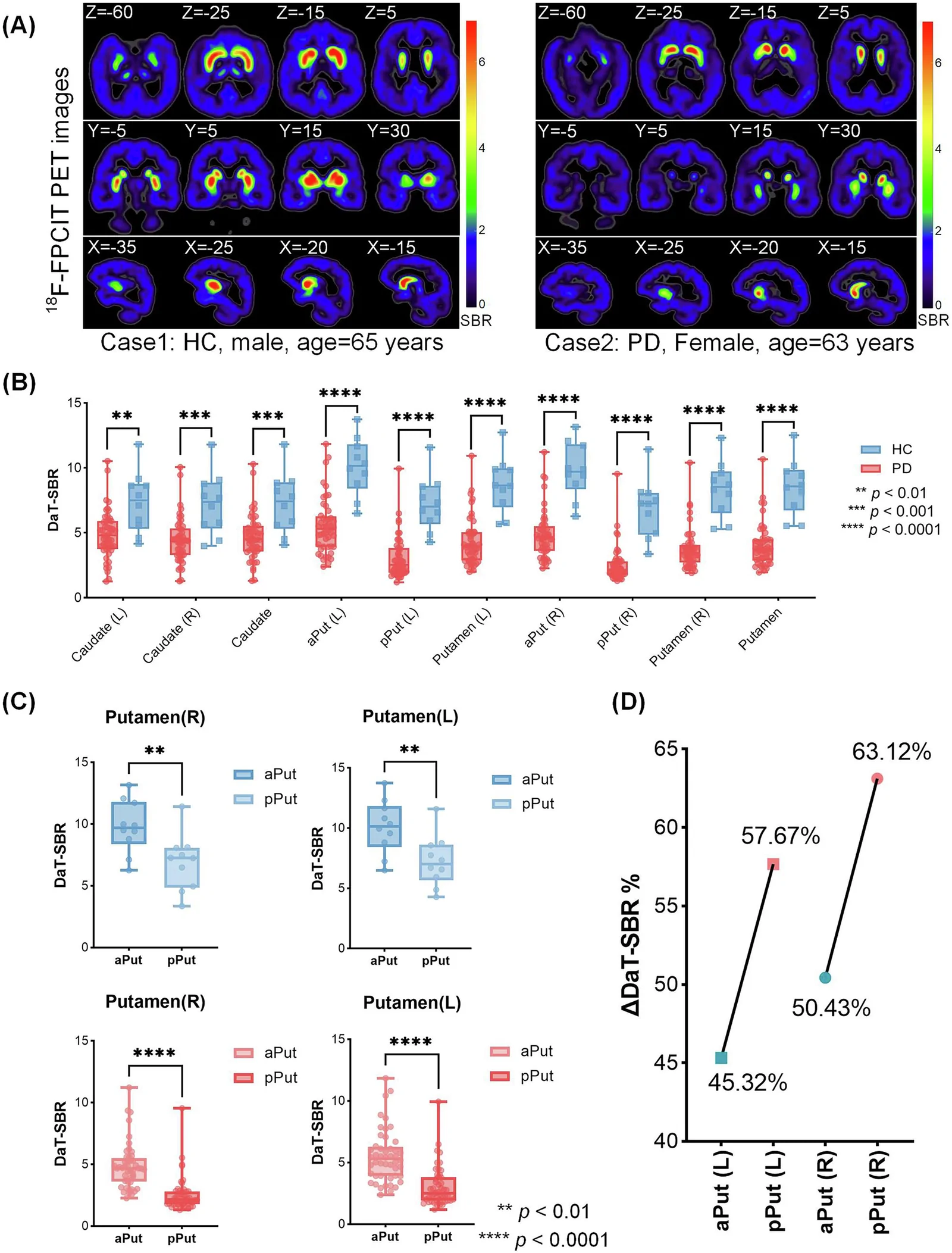
Striatal DaT-SBR reductions in PD. **A** Representative ¹⁸F-FPCIT PET image of an HC (left) and a patient with PD (right). From left to right, images correspond to different slice levels. From top to bottom, rows represent axial, coronal, and sagittal planes, respectively, illustrating preserved striatal DaT binding in the HC and marked posterior putaminal DaT loss in the PD patient. **B** Significantly lower DaT-SBR in PD patients compared to HCs across the subregions of caudate and putamen. **C** Within the putamen, DaT-SBR was significantly lower in the pPut compared to the aPut bilaterally. Data are shown for HCs (top row, blue, *n* = 10) and PDs (bottom row, red, *n* = 48). **D** Normalized DaT-SBR (HC = 100%) demonstrated a stepwise reduction, most pronounced in the pPut, followed by the aPut. aPut anterior putamen, DaT dopamine transporter, HC healthy control, L left hemisphere, PD Parkinson’s disease, PET positron emission tomography, pPut posterior putamen, R right hemisphere, SBR specific binding ratio. **p < 0.01; ***p < 0.001; ****p < 0.0001.

Representative ¹⁸F-FPCIT DaT-PET images show preserved striatal binding in HC and markedly reduced binding in PD (Fig. 1A). Groupwise comparisons demonstrated lower DaT-specific binding ratio (SBR) in PD across all striatal subregions, including caudate (left [L]: *p* = 0.0015, Cohen’s *d (d)* = 1.2858; right [R]: *p* = 0.0007, *d* = 1.4811), anterior putamen (aPut) (L/R: *p* < 0.0001, *d* = 2.1288/2.5945), posterior putamen (pPut) (L/R: *p* < 0.0001, *d* = 2.4342/2.7987), and whole putamen (L/R: *p* < 0.0001, *d* = 2.3050/2.7716) (Fig. 1B). Within the putamen, DaT-SBR was significantly lower in the pPut compared with the aPut bilaterally in both HC (*p* < 0.01) and PD (*p* < 0.0001) (Fig. 1C). In contrast, within the PD group, pairwise statistical comparisons revealed no significant difference in DaT-SBR between the caudate and the aPut (all *p* > 0.05; data not shown). Normalized to HC = 100%, PD reductions were greatest in pPut (L: 57.67%; R: 63.12%), followed by aPut (L: 45.32%; R: 50.43%) (Fig. 1D). Reductions in the caudate were more modest (L: 34.09%; R: 38.24%), as derived from normalized group means and not separately visualized (data not shown). The anatomical localization of the aPut and pPut is illustrated in Supplementary Fig. 1. Whole-brain voxelwise maps corroborated bilateral striatal reductions (Supplementary Fig. 2). In addition to striatal regions, we further examined DaT binding in the SNc. SNc FPCIT-SBR was significantly reduced in PD compared with HCs (*p* < 0.0001). However, the magnitude of reduction in the SNc was smaller than that observed in the striatum, consistent with the known distribution of dopamine transporter density, which is highest in striatal dopaminergic terminals and relatively lower in the midbrain.

### SNc iron load (QSM) and cross-species support

**Fig. 2:**
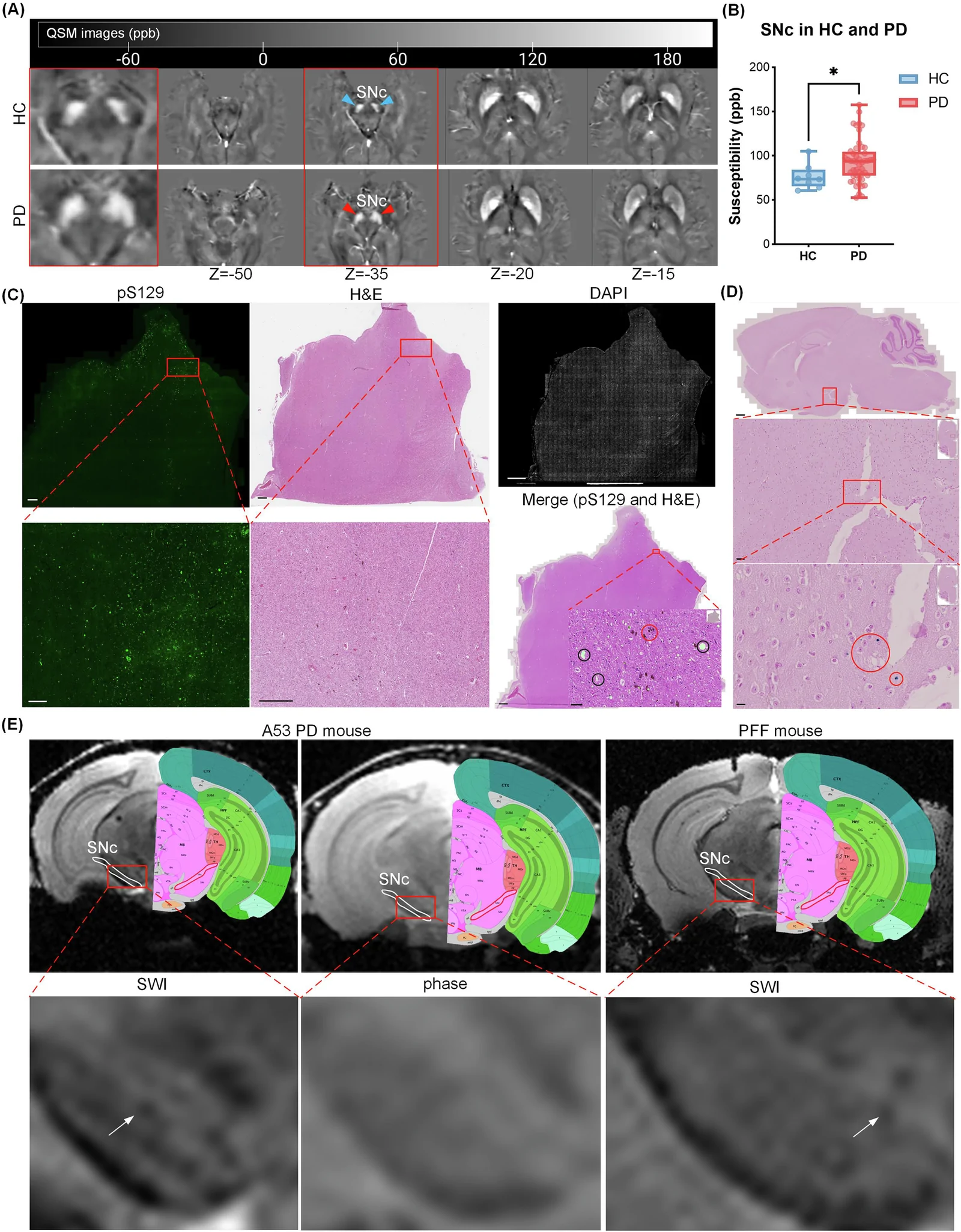
Iron deposition in the SNc of PD patients and transgenic PD mouse models. **A** Representative QSM images of an HC (top row) and a patient with PD (bottom row). Images are shown from left to right at different slice levels through the striatum and SNc. The first column shows magnified views of the SNc, corresponding to the region indicated in the third column. Brighter signal intensity indicates higher magnetic susceptibility, reflecting increased iron deposition. **B** Group-level comparisons revealed significantly greater SNc iron signals in PD patients compared with HCs (*p* = 0.0365). SNc susceptibility values were calculated at the level of the posterior limb of the internal capsule, which served as the reference region for susceptibility estimation. **C** Post-mortem H&E staining of putaminal tissue from a representative PD case revealed iron deposition (brown), while pS129 immunostaining (green) identified concomitant α-synuclein pathology; colocalization between the two signals was observed. Nuclei counterstained with DAPI (white). Scale bars: 1000 μm (row 1, all panels); 100 μm (row 2, first and second panels); 1000 μm (original image) and 50 μm (magnified view) (row 2, third panel). **D** Prussian blue staining of sagittal brain sections from an A53T transgenic PD mouse. The SNc region (red boxes) shows focal blue precipitates (red circles) at higher magnification, reflecting ferric iron deposition based on the iron-specific Prussian blue reaction. Images are shown from one representative animal and are intended for qualitative illustration only. **E** Representative ex vivo SWI (left) and corresponding phase image (middle) from an A53T transgenic PD mouse, and representative ex vivo SWI images (right) from a PFF mouse model. Images shown are from one representative animal per group. Arrows indicate focal hypointense signals within the SNc on both SWI and phase images, consistent with iron deposition. DAPI 4’, 6-diamidino-2-phenylindole, HC healthy control, H&E Hematoxylin & Eosin, PFF preformed fibril, PD Parkinson’s disease, ppb parts per billion, pS129 phospho-α-synuclein, SNc substantia nigra pars compacta, SWI susceptibility weighted imaging. *p < 0.05.

Exemplar QSM images illustrate higher SNc susceptibility in PD versus HC (Fig. 2A). At the group level, SNc susceptibility was significantly elevated in PD (*p* = 0.0365, *d* = −0.5701; Fig. 2B). Post-mortem human tissue showed iron-positive deposits on H&E with co-localized pS129 α-synuclein immunoreactivity in putaminal sections (Fig. 2C). Prussian blue staining of sagittal brain sections from A53T transgenic PD mice provided histochemical evidence for the presence of iron-containing deposits within the SNc, as indicated by focal blue staining typical of ferric iron (Fig. 2D). Representative ex vivo susceptibility weighted imaging (SWI) and phase images from a single A53T transgenic mouse demonstrated increased iron-related signal within the SNc (Fig. 2E). Similarly, representative SWI images from one α-synuclein PFF–injected mouse showed iron accumulation in the SNc (Fig. 2E). These mouse imaging data are presented for qualitative cross-species validation only.

### Cross-modal associations: SNc iron ↔ striatal DaT and microstructure

In PD, putaminal susceptibility was higher in pPut than aPut bilaterally (*p* < 0.0001; Supplementary Fig. 3A). In contrast, within the PD group, pairwise comparisons revealed no significant difference in susceptibility between the aPut and the caudate (*p* > 0.05; data not shown). Voxelwise partial correlations (adjusted for age and disease duration) revealed negative associations between SNc susceptibility and striatal DaT-SBR (Supplementary Fig. 3B).

**Fig. 3:**
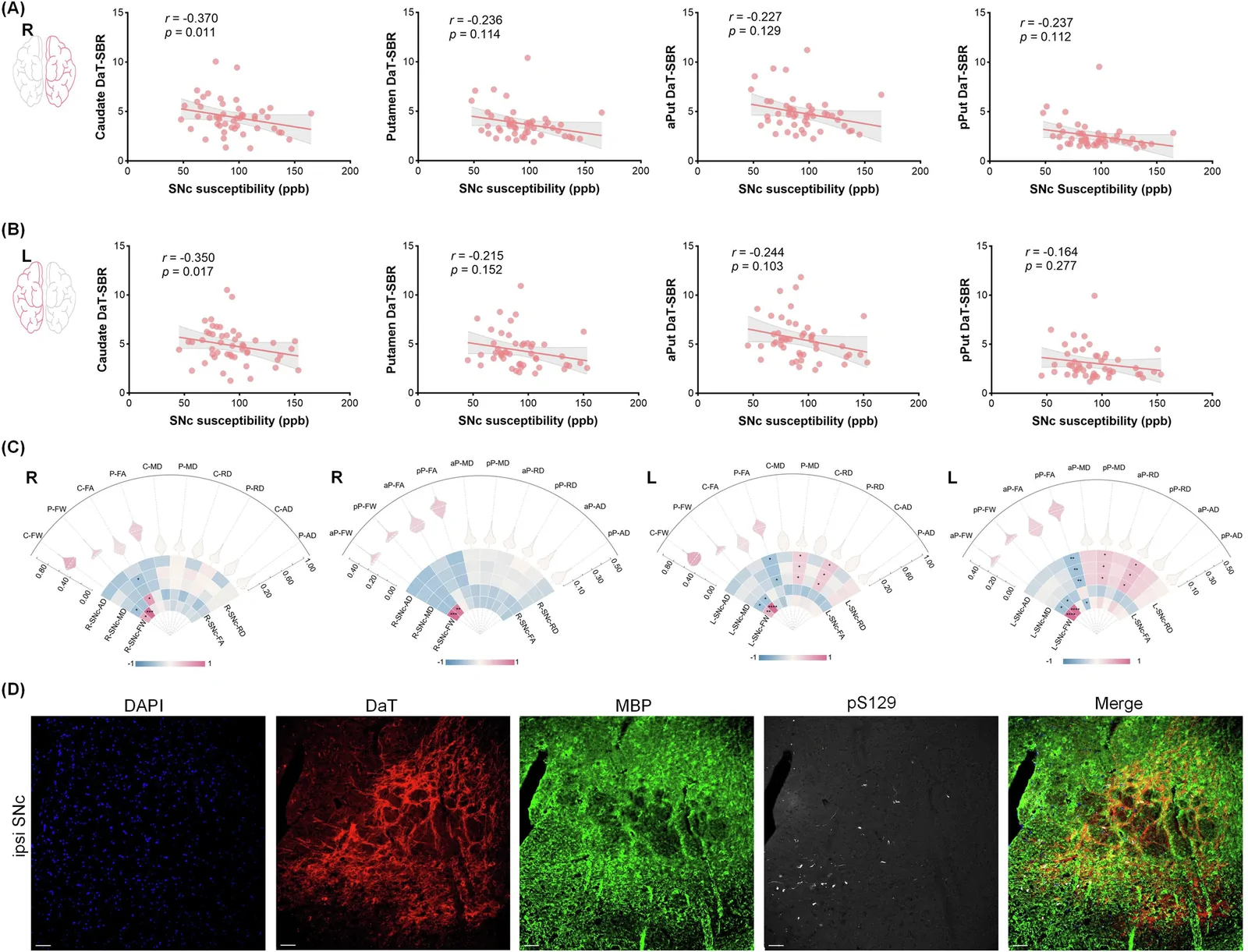
Associations between SNc pathology and striatal dysfunction in PD patients and immunohistochemical validation in a mouse model. **A** Partial correlation analysis (adjusted for age and disease duration) shows significant negative associations between right SNc susceptibility and DaT-SBR in the right caudate. **B** Significant negative correlations were observed in the left caudate. **C** Hemisphere-specific correlation matrices between SNc diffusion metrics and striatal diffusion properties in PD. From left to right, the four circular heatmaps depict: (i) correlations between right SNc diffusion metrics (MD, RD, AD, FA, FW; *x*-axis) and diffusion metrics of the right caudate and putamen (*y*-axis); (ii) correlations between right SNc diffusion metrics (*x*-axis) and diffusion metrics of the right anterior and posterior putamen (*y*-axis); (iii) correlations between left SNc diffusion metrics (*x*-axis) and diffusion metrics of the left caudate and putamen (*y*-axis); (iv) correlations between left SNc diffusion metrics (*x*-axis) and diffusion metrics of the left anterior and posterior putamen (*y*-axis). Color intensity indicates the strength and direction of the partial correlation. All data in panels. Notes: C = caudate, *P* = putamen, a*P* = anterior putamen, p*P* = posterior putamen, R = right hemisphere, L = left hemisphere. **A**–**C** represent PD patients (*n* = 48). **D** Immunohistochemistry in the brain tissue of the α-synuclein PFF mouse model revealed overlapping signals of DaT (red), MBP (green), and pS129 (white) in the ipsilateral SNc. Nuclei counterstained with DAPI (blue). All scale bars = 50 μm. AD axial diffusivity, aPut anterior putamen, DAPI 4’, 6-diamidino-2-phenylindole; DaT, dopamine transporter, FA fractional anisotropy, FW free water, MBP myelin basic protein, MD mean diffusivity, PFF preformed fibril, ppb parts per billion, pPut posterior putamen, pS129 phospho-α-synuclein, R right, RD radial diffusivity, SBR specific binding ratio, SNc substantia nigra pars compacta, Susceptibility magnetic susceptibility.

After adjusting for age and disease duration, ROI-level analyses confirmed that right SNc susceptibility was inversely correlated with right caudate DaT-SBR (*r* = -0.370, *p* = 0.011) (Fig. 3A). On the left side, significant inverse correlations were also observed between SNc susceptibility and caudate DaT-SBR (*r* = -0.350, *p* = 0.017) (Fig. 3B), whereas the associations with the putamen did not reach statistical significance. Additional sensitivity analyses further adjusting for LEDD yielded similar results, indicating that the observed associations were not substantially influenced by medication dosage.

ROI-based diffusion MRI (adjusted for age and disease duration) showed hemisphere-specific coupling between SNc and striatal microstructure. On the right hemisphere, SNc free water (FW) correlated with caudate FW (*r* = 0.312, *p* = 0.049) and putamen FW (whole: *r* = 0.518, aPut: *r* = 0.539, pPut: *r* = 0.489; all *p* < 0.001); SNc FA correlated negatively with caudate FW (*r* = -0.362, *p* = 0.022) and positively with caudate FA (*r* = 0.360, *p* = 0.023); SNc RD correlated negatively with caudate FA (*r* = -0.321, *p* = 0.043) (Fig. 3C). On the left hemisphere, SNc FW correlated positively with striatal FW—putamen (whole: *r* = 0.729, aPut: *r* = 0.733, pPut: *r* = 0.702, all *p* < 0.0001) and caudate (*r* = 0.447, *p* = 0.004), while SNc FW related negatively to pPut FA (*r* = -0.321, *p* = 0.044). SNc FA correlated negatively with striatal FW (e.g., caudate: *r* = -0.375, *p* = 0.017; putamen: *r* = -0.365, *p* = 0.020; aPut: *r* = -0.354, *p* = 0.025; pPut *r* = -0.366, *p* = 0.020) (Fig. 3C). SNc diffusivity metrics (MD, RD, and AD) showed consistent positive correlations with those in the putamen (whole and pPut; *r* range: 0.314 to 0.380, all *p* < 0.05). Conversely, these SNc metrics were negatively associated with putaminal FA, particularly in the pPut (*r* range: −0.336 to −0.457, all *p* < 0.05) (Fig. 3C). Colocalization was observed between DaT, myelin basic protein (MBP), and pS129 staining of phospho-α-synuclein deposits in the ipsilateral SNc of an α-synuclein PFF mouse model (Fig. 3D). pS129 positive phospho-α-synuclein deposits colocalized with tyrosine hydroxylase in the ipsilateral SNc (Supplementary Fig. 4), and DaT signals showed spatial overlap with MBP-labeled myelinated structures in the striatum and contralateral SNc of α-synuclein PFF-injected mice (Supplementary Fig. 5). These observations are descriptive and qualitative, intended to illustrate the anatomical proximity of pathogenic α-synuclein to dopaminergic terminals and myelinated fibers, rather than to provide quantitative evidence of axonal or myelin loss.

### Nigrostriatal tractography reveals pathway- and subregion-specific diffusion patterns

**Fig. 4:**
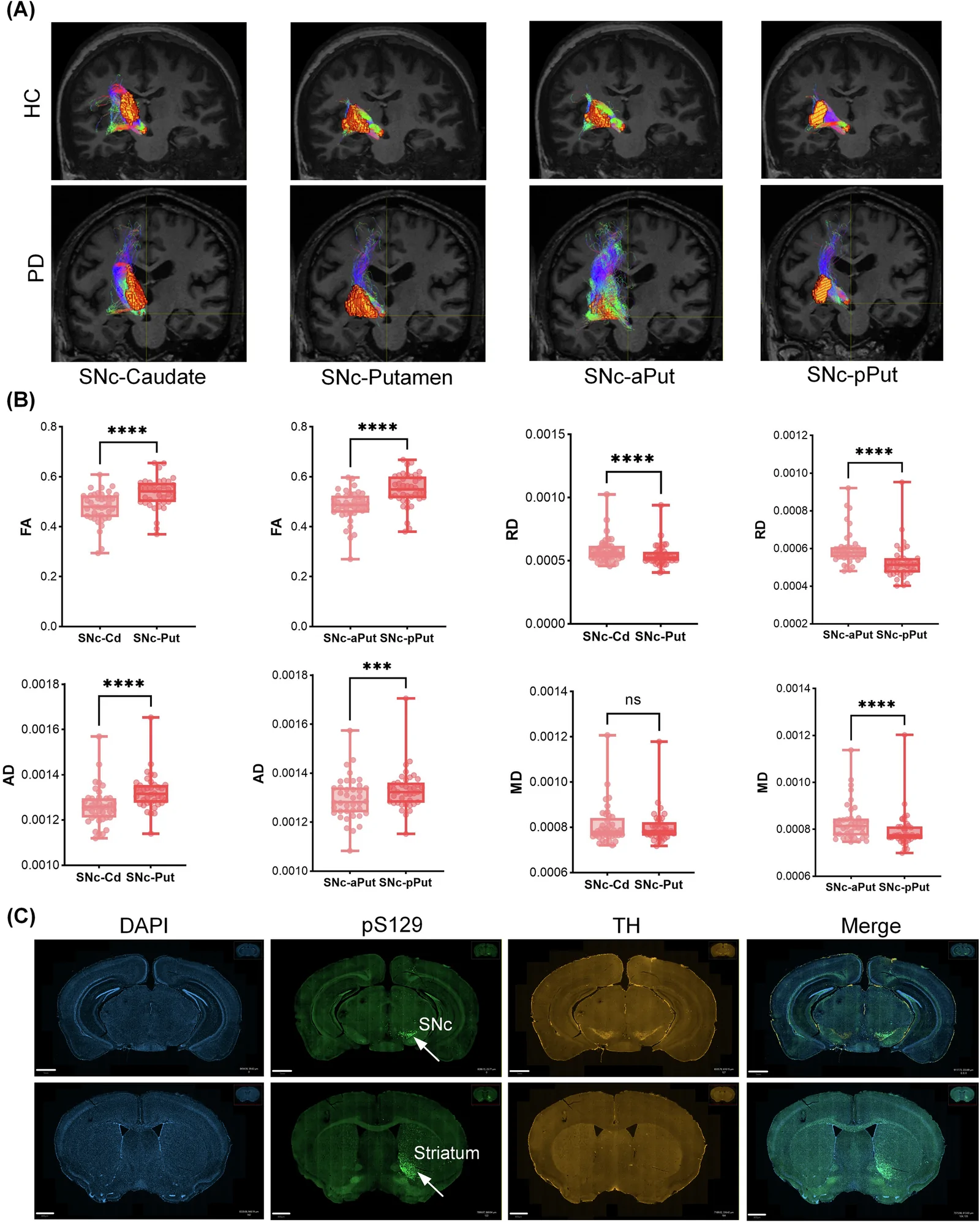
Diffusion tractography reveals subregional vulnerability of the nigrostriatal pathway in PD and supporting evidence from the α-synuclein PFF mouse model. **A** Representative DTI tractography of bilateral SNc-striatal pathways in a HC and a patient with PD, including SNc-caudate, SNc-putamen, SNc-aPut, and SNc-pPut. Fiber colors indicate the principal diffusion direction (red: left–right; green: anterior–posterior; blue: superior–inferior). **B** Group comparisons of diffusion metrics in PD patients, showing pronounced alterations in SNc-Put pathways compared with SNc-Cd, and greater vulnerability of the SNc-pPut pathway relative to the SNc-aPut pathway. All data in (**B**) represent PD patients (*n* = 48). **C** Staining of TH (yellow) and α-synuclein (green) in α-synuclein SNc-injected PFF mouse brain. pS129 signals were restricted to the ipsilateral medial striatum, 12 weeks after the unilateral injection of α-synuclein PFF into the SNc of the mouse brain. AD axial diffusivity, aPut anterior putamen, Cd caudate, FA fractional anisotropy, Nuclei counterstained with DAPI (blue). Scale bar = 1 mm (row 1) and 800 μm (row 2). AD axial diffusivity, aPut anterior putamen, Cd caudate, FA fractional anisotropy, DAPI 4’, 6-diamidino-2-phenylindole, DTI diffusion tensor imaging, MD mean diffusivity, PFF preformed fibril, PD Parkinson’s disease, pPut posterior putamen, pS129 phospho-α-synuclein, Put putamen, RD radial diffusivity, SNc substantia nigra pars compacta, TH tyrosine hydroxylase, ns not significant; ***p < 0.001; ****p < 0.0001.

Probabilistic tractography was used to delineate four nigrostriatal sub-pathways in each hemisphere: SNc→caudate, SNc→putamen, SNc→aPut, and SNc→pPut. Representative probabilistic tractography from one HC and one patient with PD is shown to illustrate the spatial organization of nigrostriatal sub-pathways (Fig. 4A and Supplementary Fig. 6B). In the tractography visualizations, fiber colors represent the principal diffusion direction (red: left–right, green: anterior–posterior, blue: superior–inferior), following standard direction-encoded color conventions. At the tract level, the representative PD subject shows visually sparser and less voluminous SNc–striatal projections than the representative HC, suggesting reduced tract coherence. These qualitative features are most apparent in SNc–putaminal pathways, particularly toward the put. At the anatomical level, the underlying T1-weighted images reveal larger ventricular spaces in representative PD cases. Tractography is visualized in each subject’s native space, and these differences reflect inter-individual anatomical variability, likely related to disease-associated cerebral atrophy, rather than tractography-derived effects. Analyses were structured hierarchically to assess (i) caudate versus putamen projections, and (ii) anterior versus posterior putaminal subdivisions.

In HCs, diffusion properties differed across nigrostriatal sub-pathways, indicating intrinsic microstructural heterogeneity. SNc→pPut exhibited lower RD than SNc→aPut bilaterally (right: *p* = 0.0156; left: *p* = 0.0078). Additionally, the SNc→putamen pathway showed higher FA than SNc→caudate in the left hemisphere (*p* = 0.0234) (Supplementary Fig. 6A).

In PD, diffusion metrics showed pronounced pathway-dependent differences (Fig. 4B and Supplementary Fig. 6C). Right hemisphere: versus SNc→caudate, SNc→putamen exhibited higher FA/AD and lower RD (all *p* < 0.0001), with no MD difference; further subdivision revealed SNc→pPut had higher FA (*p* < 0.0001)/AD (*p* = 0.0003) and lower RD/MD (all *p* < 0.0001) than SNc→aPut. Left hemisphere: a similar pattern obtained—SNc→putamen > SNc→caudate in FA/AD (all *p* < 0.0001) with lower RD (*p* = 0.0273); SNc→pPut again showed higher FA (*p* < 0.0001)/AD (*p* = 0.0345) and lower RD/MD (*p* < 0.0001; *p* = 0.0001) relative to SNc→aPut. Immunohistochemistry in the α-synuclein PFF SNc-injected mouse model provided qualitative, regionally consistent support for the imaging findings. pS129-positive phospho-α-synuclein aggregates were detected in the ipsilateral SNc and extended selectively into the medial striatum (Fig. 4C), rather than diffusely throughout the striatum. This spatially restricted propagation mirrors the non-uniform vulnerability of nigrostriatal sub-pathways observed in human PD tractography. These findings are presented as qualitative observations intended to provide anatomical and biological context for the human imaging results, rather than as formal quantitative analyses.

Notably, these FA increases and RD decreases occurred in the context of PD-related degeneration and should be interpreted as pathway-specific diffusion signatures rather than indicators of preserved microstructural integrity.

Representative LFB-stained sections from one A53T mouse and one WT mouse (*n* = 1 per group) are shown in Supplementary Fig. 7. The A53T section displays qualitatively weaker LFB staining along the SNc–striatal pathway compared with WT. These images are presented for illustrative purposes only and do not allow quantitative or statistical inference regarding myelin or axonal integrity.

### Serial mediation: SNc iron → (SNc DaT + microstructure) → striatal DaT

**Fig. 5:**
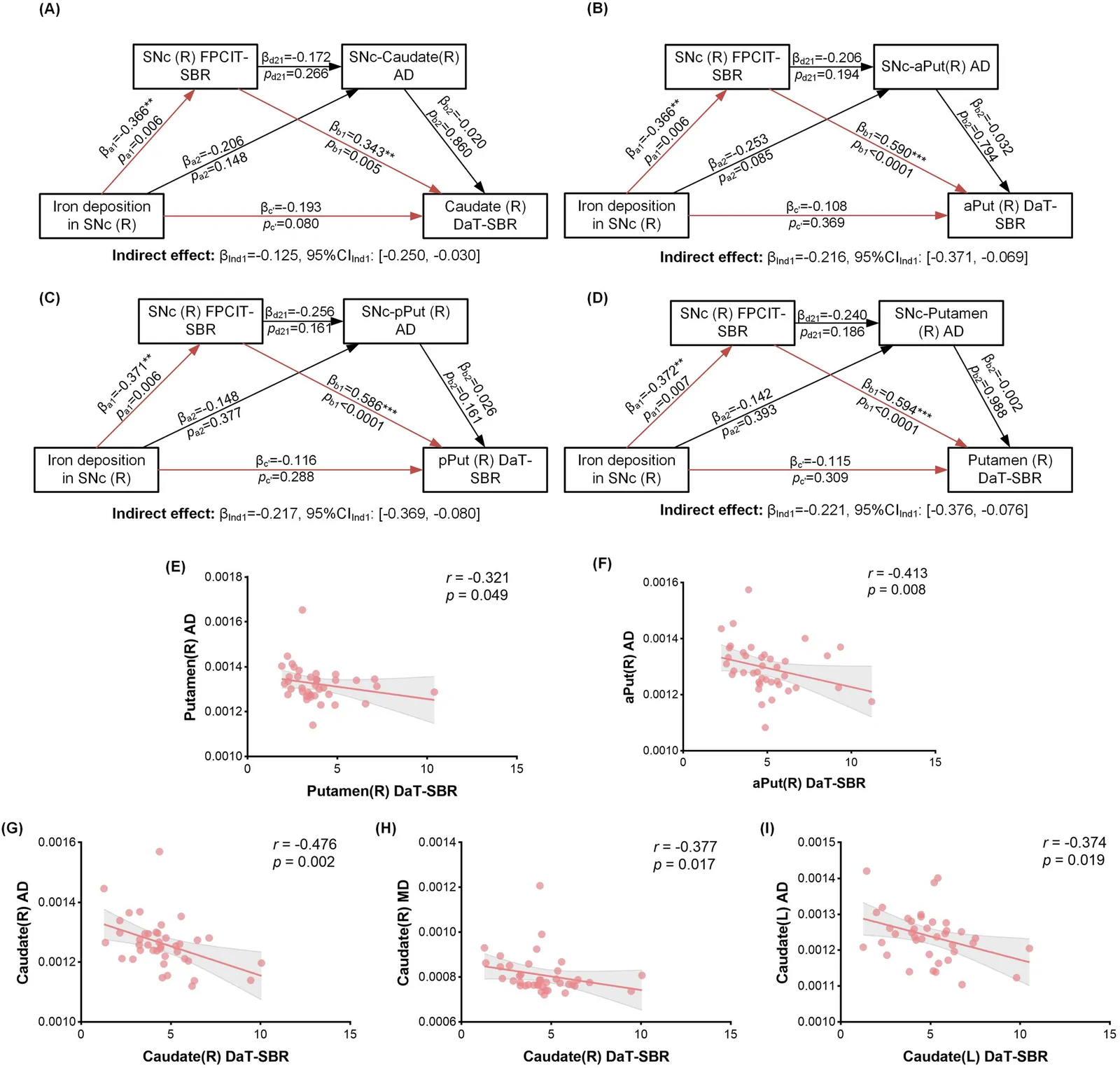
SNc iron deposition drives striatal dopaminergic dysfunction via presynaptic impairment and microstructural alterations in PD patients. **A**–**D** Serial mediation models demonstrating that increased iron deposition in the SNc exerted significant indirect effects on striatal DaT-SBR via reduced SNc FPCIT-SBR binding and microstructural deficits (FA, MD, AD, RD) in the subpathways of SNc-striatum (including SNc-caudate, SNc-aPut, SNc-pPut, and SNc-putamen). Direct effects of SNc iron on striatal DaT binding were not significant. Note: Ind1 = *a*₁ × *b*₁ (SNc iron → SNc DaT-SBR → striatal DaT-SBR); Ind2 = *a*₂ × *b*₂ (SNc iron → SNc–striatal diffusion metrics → striatal DaT-SBR); Ind3 = *a*₁ × *d*₂₁ × *b*₂ (serial mediation); direct effect = *c*’ (SNc iron → striatal DaT-SBR). **A**–**D** illustrate representative serial mediation models using AD. The reported indirect effect corresponds to Ind1 (SNc iron → SNc DaT-SBR → striatal DaT-SBR). Detailed results for all diffusion metrics and indirect pathways (Ind1, Ind2, and Ind3) are provided in Supplementary Tables 4–8. **E**–**I** Partial correlation analyses showing significant negative associations between SNc-striatum diffusivity metrics and striatum DaT-SBR, including the right putamen (*r* = -0.321, *p* = 0.049), right aPut (*r* = −0.413, *p* = 0.008), right caudate (*r* = -0.476, *p* = 0.002), right caudate MD (*r* = -0.377, *p* = 0.017), and left caudate (*r* = -0.374, *p* = 0.019). All data in (**A**–**I**) represent PD patients (*n* = 48). AD axial diffusivity, aPut anterior putamen, DaT dopamine transporter, FA fractional anisotropy, L left hemisphere, MD mean diffusivity, pPut posterior putamen, R right hemisphere, RD radial diffusivity, SBR specific binding ratio, SNc substantia nigra pars compacta.

Serial mediation models (age/sex-adjusted) tested a directed chain from SNc iron (QSM) to striatal DaT-SBR, mediated by SNc DaT-SBR (¹⁸F-FPCIT), as a proxy of nigral dopaminergic integrity, and nigrostriatal microstructure (FA/MD/AD/RD) (Fig. 5). Analyses were restricted to PD patients to examine disease-specific pathophysiological relationships. Standardized path coefficients (β) were reported for all model paths, and completely standardized indirect effects, as defined in the PROCESS macro, were calculated to quantify the magnitude of mediation effects and enable comparison across pathways.

In the right hemisphere, significant indirect effects of SNc iron on striatal DaT-SBR were observed via SNc DaT-SBR alone (Ind1: *a*₁ × *b*₁) for the caudate (β_indirect = −0.125 to −0.127; 95% CI excluding 0), aPut (−0.213 to −0.216), pPut (−0.212 to −0.221), and whole putamen (−0.219 to −0.225) (Fig. 5A–D illustrate representative models using AD, whereas results for FA, MD, and RD are summarized in Supplementary Tables 1–5). As illustrated in Fig. 5A–D, these panels present representative serial mediation models using AD, in which the direct path (c′: SNc iron → striatal DaT-SBR) is explicitly shown and was non-significant across regions. Detailed results for all diffusion metrics (AD, FA, MD, RD) and for all indirect pathways (Ind1, Ind2 and Ind3) are summarized in Supplementary Tables 2–5.

In the left hemisphere, significant Ind1 indirect effects (*a*₁ × *b*₁) linked SNc iron to left caudate DaT-SBR via SNc DaT-SBR, consistently across diffusion metrics (FA, MD, AD, RD; e.g., FA *β* = −0.099, 95% CI [ − 0.206, −0.015]; MD *β* = −0.099 [ − 0.212, −0.016]; AD *β* = −0.096 [ − 0.206, −0.012]; RD *β* = −0.101 [ − 0.211, −0.014]). The corresponding estimates can be found in Supplementary Tables 2–5. Analogous Ind1 effects were observed for aPut (*β* = −0.137 to −0.143) and pPut (β = −0.132 to −0.136).

Correlations were consistent with model paths: higher diffusivity ↔ lower DaT-SBR (e.g., right putamen AD *r* = -0.321, *p* = 0.049; right aPut *r* = -0.413, *p* = 0.008; right caudate *r* = −0.476, *p* = 0.002; right caudate MD *r* = −0.377, *p* = 0.017; left caudate *r* = −0.374, *p* = 0.019) (Fig. 5E–I).

### Region-specific diagnostic performance (ROC)

**Fig. 6:**
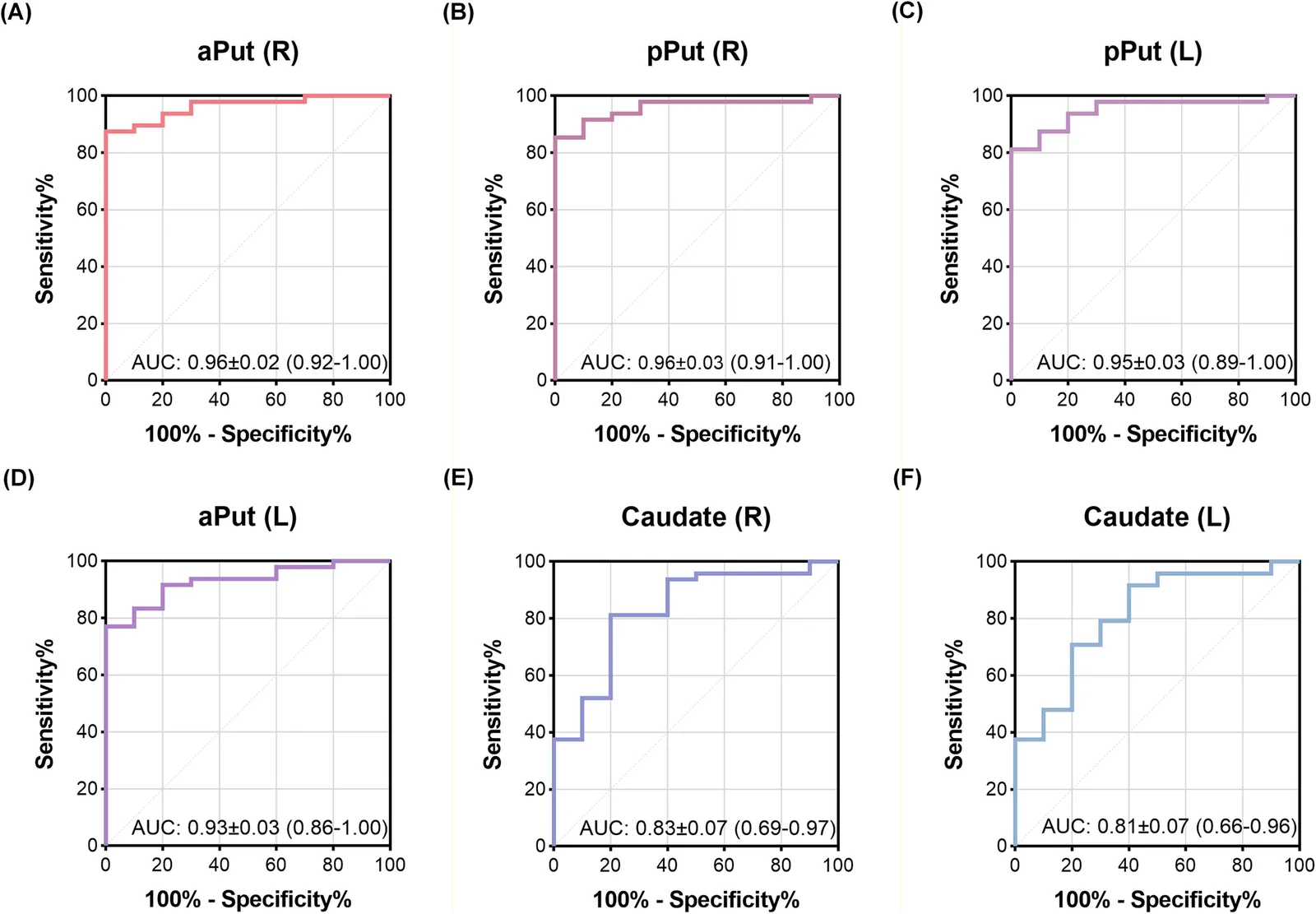
ROC analysis of DaT-SBR for discriminating PD patients from HCs. **A**–**C** ROC curves and AUC values for bilateral putamen subregions, showing near-perfect accuracy (AUCs 0.93–0.96). **D**–**F** ROC curves and AUC values for bilateral caudate regions, demonstrating lower yet robust performance (AUCs 0.81–0.83). Data are shown for HCs (*n* = 10) and PDs (*n* = 48). AUC area under the curve, DaT dopamine transporter, L left hemisphere, R right hemisphere, ROC receiver operating characteristic, SBR specific binding ratio.

ROC analyses showed excellent discrimination by putaminal DaT-SBR: right aPut AUC 0.96 ± 0.02 (95% CI 0.92–1.00), right pPut 0.96 ± 0.03 (0.91–1.00), left pPut 0.95 ± 0.03 (0.89–1.00), left aPut 0.93 ± 0.03 (0.86–1.00) (Fig. 6A–D). Caudate accuracy was lower but robust (right 0.83 ± 0.07 [0.69–0.97]; left 0.81 ± 0.07 [0.66–0.96]) (Fig. 6E, F). The AUCs for the right aPut and pPut were significantly higher than those for the right caudate (*p* = 0.023 and *p* = 0.035, respectively), and similarly, the AUCs for the left aPut and pPut were higher than those for the left caudate (*p* = 0.024 and *p* = 0.018, respectively). No significant differences were observed between aPut and pPut within each hemisphere (right, *p* = 0.676; left, *p* = 0.211). Overall, the putamen is more effective than the caudate for PD-HC discrimination.

## Discussion

Using a multimodal human–animal framework, we demonstrate that posterior-dominant putaminal dopaminergic loss in PD is not an isolated target phenomenon, but is tightly coupled to upstream SNc pathology, as indexed by QSM-derived iron burden and DTI-defined microstructural disorganization of the nigrostriatal system. Critically, serial mediation indicates that SNc iron relates to striatal DaT reductions indirectly, via reduced nigral DaT, rather than through a detectable direct path. These convergent observations support a model in which iron-linked SNc vulnerability and axonal pathway compromise together bias degeneration toward the posterior putamen, thereby explaining the robust posterior→anterior gradient observed across cohorts.

PD exhibits a characteristic posterior-to-anterior gradient of nigrostriatal dopaminergic degeneration, with preferential involvement of the posterior/dorsal putamen and relative sparing of anterior/ventral striatal regions in early disease. Importantly, degeneration of the nigrostriatal pathway is generally believed to occur earlier at striatal dopaminergic terminals than at nigral neuronal cell bodies. Accordingly, the pronounced reductions observed in the posterior putamen likely reflect early terminal degeneration, whereas the SNc DaT signal may provide complementary information regarding upstream nigral involvement. This topography has been demonstrated by post-mortem pathology^12^, DaT PET^13^, and ^18^F-DOPA PET studies^14^. Recent in vivo mapping of striatal microstructural gradients further shows that the putamen harbors robust spatial gradients that are disrupted in PD, with abnormal posterior putaminal microstructure spatially overlapping regions of dopaminergic loss and motor dysfunction^4^. Together, these converging molecular and microstructural findings support a gradient-based framework for understanding posterior putaminal vulnerability in PD.

We observed elevated SNc susceptibility in PD and negative associations between SNc iron and striatal DaT-SBR, most consistently in the put. These findings are consistent with experimental and pathological evidence indicating that intracellular iron interacts closely with α-synuclein biology and cellular redox pathways, thereby increasing the vulnerability of nigrostriatal dopaminergic neurons^15,16^. Notably, progressive iron accumulation in the SNc has been shown to correlate with striatal dopaminergic denervation and neuronal loss across disease stages, supporting a tight coupling between nigral iron burden and downstream dopaminergic dysfunction^17^. In addition, converging neuroimaging studies demonstrate preferential iron accumulation along the nigrostriatal system in aging and PD, with consistently higher iron levels across multiple SN subdivisions in PD compared with controls^18^. Together, our observation of elevated iron in the posterior putamen—the epicenter of early dopaminergic depletion—supports spatial colocalization of iron changes with neurodegeneration^18^, consistent with a model in which SNc iron burden contributes to region-specific striatal dysfunction.

We observed hemisphere-specific coupling between SNc and striatal diffusion metrics, with strong SNc–putamen FW correlations and consistent inverse associations between striatal diffusivity (AD/MD/RD) and DaT-SBR. Although overt striatal neuronal loss is limited, increased diffusivity and free water likely reflect axonal and synaptic alterations accompanying dopaminergic denervation, particularly in the posterior putamen^2^. The negative diffusivity–DaT-SBR relationships further support a close link between microstructural injury and terminal dopaminergic loss, consistent with region-specific vulnerability of the nigrostriatal system in PD.

In PD, the SNc→pPut pathway exhibits a distinct diffusion profile—elevated FA and AD alongside reduced RD and MD—compared to other nigrostriatal projections. While this may intuitively suggest preserved microstructural integrity, a nuanced interpretation is required. The value of FA as a diagnostic biomarker is limited by its high between-study heterogeneity^8^. Furthermore, AD and RD are interdependent, especially in regions with complex fiber architecture or mixed pathology, meaning a change in one can spuriously influence the other^9^. Therefore, these DTI metrics should not be interpreted in isolation as direct markers of isolated axonal or myelin change^19^. Notably, striatal neuronal loss is limited in PD, and diffusion changes—particularly in AD—may reflect regression of dopaminergic axons and synaptic terminals rather than neuronal soma loss^20^. Instead, this specific pattern likely reflects pathway-selective microstructural reorganization resulting from degeneration. Selective loss of crossing fibers or specific neuronal subpopulations could increase the relative coherence of remaining tissue, elevating FA. Concurrent changes in tissue density and composition may drive the observed AD and RD shifts. This aligns with established models of posterior-to-anterior striatal degeneration in PD and underscores the differential vulnerability within the nigrostriatal system^2^. Future studies using advanced diffusion models are warranted to better resolve the underlying pathophysiology.

Qualitative histopathological observations in the α-synuclein PFF SNc-injected mouse model provide convergent, but non-quantitative, support for this interpretation. The selective presence of pS129-positive aggregates in the ipsilateral SNc and medial striatum suggests that pathogenic α-synuclein does not propagate uniformly along all nigrostriatal projections, but instead follows anatomically and functionally vulnerable pathways^21,22^. This spatial restriction is consistent with the pathway-dependent diffusion signatures observed in vivo, rather than serving as a direct validation of diffusion metrics. Furthermore, the colocalization of pS129 with DaT and MBP highlights the close anatomical relationship between α-synuclein pathology, dopaminergic terminals, and myelinated axons^6,23^.

Our serial mediation findings indicate that SNc iron relates to striatal DaT loss indirectly through nigral DaT reduction, with direct paths being non-significant. This pattern is consistent with, though not proof of, a sequential injury process in which iron-linked insults first impair nigral compartments/axons, and subsequently manifest as posterior-dominant terminal denervation^24^. Given the cross-sectional design, these inferences depend on assumptions of temporal ordering and the absence of unmeasured confounding; however, the human–animal convergence and topographic specificity bolster mechanistic plausibility.

The region-specific diagnostic performance of putaminal DaT-SBR (AUC ≈ 0.93–0.96)—surpassing caudate metrics (AUC ≈ 0.81–0.83)—supports the premise that posterior putaminal measures capture early, and offer high discriminative value for screening/stratification and progression monitoring. These results advocate region-prioritized readouts and provide a rationale for trial enrichment targeting iron handling or axonal integrity.

This study has several limitations. The number of healthy controls was modest, reflecting challenges in recruiting volunteers for dopamine transporter PET imaging due to radiation exposure, cost, and ethical considerations. The cross-sectional design limits temporal inference, and vascular comorbidities and partial-volume effects may confound estimates. Larger multi-center longitudinal studies will be important to improve statistical power, reproducibility, and generalizability. Given the intrinsic asymmetry of PD, hemisphere standardization was used solely to align disease severity across patients with heterogeneous symptom laterality and improve group-level sensitivity, rather than to infer true hemispheric effects^25^. Importantly, the principal group differences in striatal DaT-SBR remained significant when analyses were performed without hemisphere flipping, confirming the robustness of our findings. QSM and DTI have model-specific sensitivities; broader adoption of free-water/biophysical models, test–retest reliability, and harmonized multi-site pipelines will improve specificity and generalizability. Future work should pursue longitudinal imaging to test whether SNc iron/microstructure predicts subsequent DaT decline in the pPut, as well as interventional trials that modulate iron metabolism or axonal health to evaluate biomarker responsiveness. Finally, the present study did not examine territory-specific associations between functional subdivisions of the SNc and striatal dopaminergic dysfunction. Future studies incorporating refined functional or connectivity-based parcellation of the SNc and striatum may help further elucidate territory-specific relationships within the nigrostriatal axis.

In conclusion, the SNc iron burden and nigrostriatal microstructural degeneration together provide a mechanistic substrate for the posterior-to-anterior gradient of striatal dopaminergic dysfunction in PD. By integrating DaT-PET, QSM, and DTI/tractography with cross-species evidence and serial mediation, we outline a biologically coherent pathway from iron-linked nigral vulnerability to posterior putaminal denervation. These insights support the development of region-specific imaging biomarkers for early detection and stratification, and motivate the establishment of therapeutic strategies targeting iron handling and axonal integrity through longitudinal and interventional studies.

## Methods

### Participants

The workflow detailing participant recruitment, exclusion criteria, and imaging quality control is presented in Supplementary Fig. 8. A total of 48 PD patients and 10 HCs were ultimately enrolled in this retrospective study conducted at Huashan Hospital, Fudan University (January 2023–April 2024). Inclusion criteria: (1) PD diagnosis independently confirmed by two movement-disorder neurologists per 2015 MDS Clinical Diagnostic Criteria; (2) ability to complete Hoehn–Yahr, UPDRS-III, and MMSE; (3) compliance with ¹⁸F-FPCIT PET/MRI; (4) written informed consent. Exclusion criteria: (1) secondary Parkinsonism/atypical syndromes; (2) severe cardiopulmonary/metabolic/psychiatric/systemic disease; (3) cerebrovascular disease on neuroimaging; (4) history of traumatic brain injury, brain tumor, or epilepsy. Ten sex- and age-matched HCs were recruited during the same period. PD participants completed UPDRS-III and MMSE before PET/MRI. Among the 48 PD patients, disease severity was distributed as follows: 6 at Hoehn-Yahr stage 1, 17 at stage 2, 18 at stage 3, 5 at stage 4, and 2 at stage 5. This study was conducted in accordance with the Declaration of Helsinki and was approved by the Institutional Review Board (or Ethics Committee) of Huashan Hospital, Shanghai, China (approval number: KY2023712). Written informed consent was obtained from all participants.

### PET/MRI imaging acquisition

All imaging was performed using an integrated 3.0T PET/MR scanner (uPMR 790, United Imaging Healthcare, Shanghai, China) with a 24-channel head coil. PD patients discontinued antiparkinsonian medication for 12 h before scanning. ¹⁸F-FPCIT (3.7 MBq/kg) was administered 90 min before scanning via the dorsal hand vein. Participants lay supine, eyes closed, with motion minimized.

PET data were acquired in list-mode over a single-bed list-mode acquisition for 45 min. Reconstruction: slice thickness 2 mm, field of view (FOV) 300 × 300 mm², 4 iterations/20 subsets, matrix 192 × 192, full width at half maximum (FWHM) 3 mm, voxel 1.56 × 1.56 × 2 mm³.

MRI data were acquired simultaneously during PET acquisition and included a 3D T1-weighted spoiled gradient-recalled echo (SPGR) sequence (repetition time [TR] 7.9 ms, echo time [TE] 2.8 ms, matrix 256 × 256, 176 slices, 1 mm thickness, FOV 256 × 256 mm²), a multi-echo gradient echo sequence for QSM (TR 28.3 ms, TE 3.4 ms, matrix 256 × 256, 118 slices, 1 mm thickness, FOV 256 × 256 mm²), and DTI using echo-planar imaging (64 directions, *b* = 0/1000 s/mm², TR 8632 ms, TE 84.9 ms, matrix 92 × 96, 60 slices, 2.5 mm thickness, FOV 230 × 240 mm²).

A comprehensive summary of all sequence parameters for the uPMR790 (3T) scanner is provided in Supplementary Table 6.

### Data processing

PET data were processed using SPM12 (https://www.fil.ion.ucl.ac.uk/spm/). PET was co-registered to native T1; T1 was segmented (GM/WM/CSF); partial-volume correction (PVC) was performed using the PETPVE12 toolbox based on tissue priors^26^; images were smoothed (8 mm FWHM). DaT-SBR was computed using the occipital cortex as the reference region. Given the relatively low intrinsic DaT signal in the midbrain, SNc DaT-SBR should be interpreted as a relative index of nigral dopaminergic neuronal integrity rather than a primary quantitative marker of terminal density^27^. Partial-volume correction and anatomically constrained ROIs were applied to mitigate spill-in and susceptibility effects. SNc DaT-SBR was used exclusively in mediation analyses to model upstream nigral involvement and was not used for diagnostic discrimination.

QSM images in parts per billion (ppb) units obtained during the scanning process were co-registered to T1 via magnitude images using SPM/FSL/ANTs. Susceptibility values were referenced to the posterior limb of the internal capsule (PLIC) due to its stable magnetic susceptibility^25^; normalized maps were smoothed using SPM12 (1 mm FWHM).

DTI preprocessing followed FSL v6.0.3 protocols^28,29^: TOPUP (susceptibility correction), BET (brain extraction), EDDY (motion/eddy-current with outlier replacement), then DTIFIT to derive FA/MD/AD/RD. The eddy-corrected diffusion-weighted images and corresponding B0 images were used to estimate voxel-wise free water (FW), reflecting the fractional volume of extracellular water, using the DIPY software package. FW metrics were extracted from SNc and striatal ROIs, whereas FW was not modeled along the reconstructed nigrostriatal tracts.

Probabilistic tractography was conducted using MRtrix3. Response functions for spherical deconvolution and fiber orientation distributions (FOD) were estimated using dwi2response and dwi2fod, respectively, with a maximum spherical harmonic order (*l*max) of 8. Streamline generation used iFOD2 with streanmline number = 5000, seeds number = 1000 × streamline number, step = 0.5× voxel, angle = 45°, cutoff = 0.1, minlength = 20 mm, maxlength = 200 mm; seed–target masks defined SNc→caudate/putamen and SNc→aPut/pPut sub-pathways.

Bilateral caudate and putamen were segmented using FreeSurfer. Putamen masks were subdivided into aPut and pPut using a predefined anatomical boundary along the coronal plane by an equal-distance segmentation approach. The SNc ROI originated from a neuromelanin-sensitive MRI template^30^, which was transformed into native T1 space using ANTs. For each ROI, we extracted DaT-SBR and QSM; DTI metrics were summarized per ROI/tract. ROI-based analyses were performed within anatomically defined gray matter nuclei (SNc and striatum), whereas tract-based analyses were conducted along reconstructed nigrostriatal projection pathways derived from probabilistic tractography. To address the inherent symptom laterality in PD and to augment statistical power for group-level comparisons, a hemisphere standardization procedure was applied for all primary analyses^25^. For lateralized PD patients, imaging data were oriented so that the hemisphere contralateral to the clinically more affected body side corresponded to the left side. Data from non-lateralized PD patients and healthy controls were not flipped. We acknowledge that hemisphere flipping has methodological limitations and may obscure genuine hemispheric differences. Therefore, to confirm that the principal findings were not driven by this standardization procedure, we performed a complementary analysis without hemisphere flipping. Specifically, bilateral striatal DaT-SBR derived from unflipped ^18^F-FPCIT PET images in PD patients and HCs were directly compared. This analysis yielded consistent and significant group differences, supporting the robustness of our main results independent of hemisphere standardization (Supplementary Fig. 9).

### Post-mortem human brain tissue

Tissue from one PD and one control donor (Supplementary Table 7) was obtained from the Netherlands Brain Bank with consent, and procedures complied with the Declaration of Helsinki and VU Medical Center approvals^31^.

### Animal models

Mouse model imaging and histopathological analyses were performed for qualitative validation only, and no quantitative statistical comparisons were conducted.

Two male C57BL/6J mice (8 weeks old) received unilateral stereotactic injections of α-synuclein PFFs into the SNc, as previously described^31^. Briefly, mice were anesthetized and placed in a stereotaxic frame, and 550 nL of α-synuclein PFF solution (2.5 µg/µL) was injected at the following coordinates relative to bregma: anteroposterior (AP) −3.0 mm, mediolateral (ML) + 1.2 mm, dorsoventral (DV) −4.4 mm. Injections were performed at a slow rate using a Hamilton syringe, and the needle was left in place for several minutes before withdrawal to prevent reflux. Mice were sacrificed 12 weeks post-injection for ex vivo MRI and immunohistochemical analyses.

In addition, transgenic mice overexpressing the human α-synuclein gene carrying the A53T mutation under the control of the mouse prion protein promoter (M83 line; mixed C57BL/6 × C3H background) were used. A53T mice (10–11 months of age, *n* = 6) and age-matched non-transgenic littermates (NTLs; both sexes, *n* = 6) were housed under standard conditions. NTLs were included as internal reference controls to ensure comparable environmental and experimental conditions and to confirm the specificity of imaging and histopathological observations. However, NTLs were not subjected to quantitative analysis or statistical comparison and are therefore not shown in the results or figures. Perfusion and anesthesia were performed according to institutional protocols. Brains were fixed in 4% paraformaldehyde and cryoprotected in 30% sucrose prior to storage at −80 °C. All procedures complied with the Swiss Federal Act on Animal Protection and were approved by the Cantonal Veterinary Office. PFF-related procedures were approved by the Regierungspräsidium Giessen (V54–19c2015h01GI20/28).

### Ex vivo MRI of mouse brains

The heads were placed in a 15 mL centrifuge tube filled with perfluoropolyether (Fomblin Y, LVAC 16/6, average molecular weight 2700, Sigma-Aldrich, USA). MRI data were acquired on a BioSpec 94/30 with a cryogenic 2 × 2 radio frequency phased-array surface coil (overall coil size 20 × 27 mm^2^, Bruker BioSpin AG, Fällanden, Switzerland) with a coil system operating at 30 K (Bruker BioSpin AG, Fällanden, Switzerland) for reception used in combination with a circularly polarized 86 mm volume resonator for transmission. For SWI, a global and MAPSHIM protocol with a field map (default settings) was used for shimming^32^. A 3D gradient-recalled echo SWI sequence was recorded with the following parameters: FOV = 15 × 12 × 15 mm; image size = 248 × 199 × 36 μm, resulting in a spatial resolution = 60 × 60 × 438 μm. One echo with a echo time = 12 ms; repetition time = 250 ms; flip angle = 15°; number of averages = 4, acquisition scan time = 1 h 59 min 24 s. SWI and phase images were computed using the SWI processing module in ParaVision 6.0.1 (Bruker, Ettlingen, Germany) with Gauss broadening = 1 mm and mask weighting = 4. All SWI images were compared with their phase image counterparts. MRI images were analyzed in ITK SNAP and evaluated by two people blinded to the genotype of the mice^33^.

### Ex vivo staining of human and mouse brain tissue

H&E was performed on 3 µm sections (human/mouse). LFB was performed on 6 µm mouse sections (overnight at 60°C) to assess myelin. Prussian blue was used to assess iron in A53T and WT mice^34^. For immunohistochemistry, human FFPE sections (3 µm) underwent deparaffinization, rehydration, and retrieval in citrate buffer (pH 6.0) at 98°C for 20 min. Mouse brains (OCT-embedded) were coronally sectioned 30–40 µm (Leica CM3050 S) and stored in cryoprotectant (ethylene glycol:glycerol:0.1 M PBS = 1:1:3) at 4°C. Primary antibodies included pS129 (Abcam), DaT, TH, and MBP (Supplementary Table 8); nuclei were counterstained with 4’, 6-diamidino-2-phenylindole (DAPI)^35^. Whole-section imaging used Axio Observer Z1 (20×); high-resolution/co-localization used Leica SP8 (20×/63×). Quantification was performed using QuPath and ImageJ; anatomical references were based on the Allen human/mouse atlases.

### Statistical analysis

Demographic categorical variables (e.g., sex) are presented as frequencies and compared using *χ*² tests. Continuous variables (e.g., age) are expressed as mean ± standard deviation and analyzed with independent two-sample *t*-tests. Effect sizes for group comparisons were quantified using Cohen’s *d*. Normality of continuous variables was assessed using the Shapiro–Wilk test and visual inspection of histograms.

Group differences in striatal DaT-SBR and SNc iron deposition between PD patients and HCs were assessed using Mann-Whitney U tests when the normality assumption was not met. Partial correlation analyses, controlling for age and disease duration, were conducted to evaluate the relationship between SNc iron and striatal DaT-SBR. Between-group differences in nigrostriatal pathway metrics were examined using independent two-sample t-tests, and correlations between SNc and striatal diffusion parameters were also assessed using Pearson correlation analyses when data were approximately normally distributed.

Mediation analyses were performed using a serial multiple mediation model (Model 6) implemented in the SPSS PROCESS macro to test whether nigrostriatal microstructural alterations sequentially mediate the relationship between SNc iron deposition and striatal DaT binding. SNc iron (QSM) was specified as X, SNc FPCIT-SBR as M1, SNc striatal diffusion metrics as M2, and striatal DaT-SBR as Y, with age and sex included as covariates. Standardized path coefficients (*β*) were estimated for each individual regression path in the serial mediation model, including *a*₁ (SNc iron → SNc DaT-SBR), *a*₂ (SNc iron → SNc–striatal diffusion metrics), *d*₂₁ (SNc DaT-SBR → SNc–striatal diffusion metrics), *b*₁ (SNc DaT-SBR → striatal DaT-SBR), *b*₂ (SNc–striatal diffusion metrics → striatal DaT-SBR), and the direct effect *c*′ (SNc iron → striatal DaT-SBR). Based on these path coefficients, three predefined indirect effects were computed using the PROCESS macro: Ind1 (SNc iron → SNc DaT-SBR → striatal DaT-SBR), quantified as *a*₁ × *b*₁; Ind2 (SNc iron → SNc–striatal diffusion metrics → striatal DaT-SBR), quantified as *a*₂ × *b*₂; and Ind3 (serial mediation via SNc DaT-SBR and diffusion metrics), quantified as *a*₁ × *d*₂₁ × *b*₂. The conceptual framework and path structure of the serial mediation model, including the definitions of direct and indirect effects, are illustrated in Supplementary Fig. 10. Serial mediation analyses were conducted in PD patients only, as HCs did not exhibit sufficient variability in nigrostriatal degeneration measures required for mediation modeling. The significance of indirect effects was tested using a nonparametric bootstrap approach with 5000 resamples, and 95% bias-corrected confidence intervals (CIs) were estimated to assess mediation effects given the non-normal sampling distribution of indirect effects. All mediation paths were predefined a priori based on neurobiological hypotheses rather than data-driven model selection.

ROC analysis was used to evaluate the diagnostic performance of regional DaT-SBR, and the DeLong test was applied to compare differences in the AUC between groups. Mouse model imaging and histopathological analyses, as well as post-mortem human tissue analyses, were performed for qualitative and cross-species validation purposes only due to limited sample sizes; no quantitative statistical comparisons were conducted on these data. All analyses were conducted in SPSS version 27.0 (IBM Corp.), with a two-sided *p* < 0.05 considered statistically significant. Given that the primary analyses were hypothesis-driven and restricted to a limited number of predefined regions of interest, no global correction for multiple comparisons was applied.

## Supplementary information

Supplementary-clean

## Data Availability

All research data supporting this publication are directly available within this publication.
