# Supplementary material for: Multimodal neuroimaging elucidates the posterior-to-anterior gradient of nigrostriatal degeneration in Parkinson’s disease
Source: NPJ Parkinsons Dis. 2026 Jul 1;12:257. doi: 10.1038/s41531-026-01456-y (PMC13686981; doi:10.1038/s41531-026-01456-y)
Supplement: Supplementary file 1 [file 41531_2026_1456_MOESM1_ESM.docx]

**Supplementary information**

**Multimodal Neuroimaging Elucidates the Posterior-to-Anterior Gradient of Nigrostriatal Degeneration in Parkinson’s Disease**

Huamei Lin, Ying Zhang, Yixin Zhao, Yiman Wei, Benjamin F. Combes, Martin T. Henrich, Fanni F. Geibl, Wolfgang H. Oertel, Kuangyu Shi, Axel Rominger, Ping Wu, Jingjie Ge, Jing Wang, Jiaying Lu, Zizhao Ju, Qian Xu, Huiwei Zhang, Yunhao Yang, Zengping Lin, Yang Yang, Yihui Guan, Fengtao Liu, Jian Wang, Jiehui Jiang, Ruiqing Ni, Chuantao Zuo

***Corresponding authors**:

Jiehui Jiang, jiangjiehui@shu.edu.cn.

Ruiqing Ni, ni@biomed.ee.ethz.ch.

Chuantao Zuo, zuochuantao@fudan.edu.cn.

**Supplementary Fig. 1 | Visualization of the anterior and posterior putamen.**

**Supplementary Fig. 2 | Voxel-wise analysis of striatal dopaminergic dysfunction in PD.** Voxel-wise analysis revealed widespread reductions of DaT-SBR in the bilateral caudate and putamen in patients with PD compared with HC. DaT, dopamine transporter; HC, healthy control; PD, Parkinson's disease; SBR, specific binding ratio.

**Supplementary Fig. 3 |** **Subregional iron susceptibility and its association with striatal DaT binding in PD**. (A) Subregional comparison of putaminal susceptibility, showing significantly higher iron levels in the pPut compared with the aPut bilaterally (*p* < 0.0001). No caudate–aPut comparison is shown in this panel. (B) Voxel-wise analysis showing significant negative correlations between SNc susceptibility and striatal DaT-SBR in the caudate and putamen after adjusting for age and disease duration. Blue clusters indicate voxels with statistically significant negative correlations (higher SNc susceptibility associated with lower DaT-SBR). Red outlines delineate the anatomical boundaries of the striatum for visualization purposes only and do not represent statistical effects or correlation direction. aPut, anterior putamen; DaT, dopamine transporter; ppb, parts per billion; pPut, posterior putamen.

**Supplementary Fig. 4 |** **Colocalization of pS129 and TH in the ipsilateral SNc of α-synuclein PFF-injected mice**. Immunofluorescence staining demonstrates colocalization of pS129 (green) within TH-positive dopaminergic neurons (yellow) in the ipsilateral SNc. From top to bottom, images are shown at progressively lower magnification, illustrating pS129–TH colocalization from the cellular level to the tissue level. Nuclei are counterstained with DAPI (blue). DAPI, 4’, 6-diamidino-2-phenylindole; PFF, preformed fibril; pS129, phospho-α-synuclein; TH, tyrosine hydroxylase.

**Supplementary Fig. 5 | Spatial overlap of DaT and MBP signals in the striatum and contralateral SNc of α-synuclein PFF-injected mice**. Representative immunofluorescence images showing overlapping spatial distribution of DaT (red) and MBP (green) signals within the striatum and contralateral SNc. Merged images illustrate the close anatomical proximity between dopaminergic elements and myelinated structures in regions affected by PFF-induced pathology. These images are intended for qualitative illustration only and are not used for quantitative colocalization analysis. DaT, dopamine transporter; MBP, myelin basic protein; PFF, preformed fibril; SNc, substantia nigra pars compacta.

**Supplementary Fig. 6 |** **Subregional comparisons of nigrostriatal pathways in HC and PD**. (A) In healthy controls, the SNc–pPut pathway showed lower RD than the SNc–aPut bilaterally, while the SNc–Put pathway exhibited higher FA than the SNc–Cd in the left hemisphere. (B) Representative tractography illustrating SNc–striatal sub-pathways in HC and PD. Fiber colors encode principal diffusion orientation (red: left–right; green: anterior–posterior; blue: superior–inferior). (C) In patients with PD, diffusion metrics revealed significant alterations across nigrostriatal sub-pathways, with disproportionately greater changes in the SNc–pPut pathway compared with the SNc–aPut. AD, axial diffusivity; aPut, anterior putamen; Cd, caudate; FA, fractional anisotropy; DTI, diffusion tensor imaging; HC, healthy control; MD, mean diffusivity; PD, Parkinson's disease; pPut, posterior putamen; Put, putamen; RD, radial diffusivity; SNc, substantia nigra pars compacta. ns, not significant; **p* < 0.05; ***p* < 0.01; ****p* < 0.001; *****p* < 0.0001.

**Supplementary Fig. 7 |** **Representative LFB-stained sections from one A53T mouse and one WT mouse (n = 1 per group)**. (A) Allen mouse brain atlas sagittal view showing the anatomical structure of the mouse brain. (B~C) Representative Luxol fast blue (LFB)–stained sagittal sections from a WT control mouse (B) and an A53T transgenic PD mouse (C), shown for anatomical illustration only. Arrows indicate corresponding anatomical regions across sections, as guided by the atlas in panel A. These images are not intended to highlight regional differences in LFB optical density and were not used for quantitative or statistical comparisons of myelin or axonal integrity. LFB, Luxol fast blue; PD, Parkinson's disease; SNc, substantia nigra pars compacta.

**Supplementary Fig. 8 | Workflow of participant recruitment, exclusion criteria, and imaging quality control**.

**Supplementary Fig. 9 | Robustness of striatal DaT-SBR group differences without hemisphere standardization**. Significant reductions in striatal DaT-SBR were observed in PD relative to HCs, consistent with the results obtained using hemisphere-standardized analyses. aPut, anterior putamen; DaT, dopamine transporter; HC, healthy control; L, left hemisphere; PD, Parkinson's disease; pPut, posterior putamen; R, right hemisphere; SBR, specific binding ratio. ***p* < 0.01; ****p* < 0.001; *****p* < 0.0001.

**Supplementary Fig. 10 | Conceptual framework and mediation effects of left SNc iron deposition on striatal DAT-SBR via SNc FPCIT-SBR and diffusion metrics**. Numerical results are provided in Supplementary Tables 4~8. Model: X=SNc-Iron, Y= Striatum DaT-SBR, M1= SNc FPCIT-SBR, M2= SNc-Striatum DTI metrics. Covariate: age and sex. (Standardized coefficients and completely standardized indirect effects in PD and HC groups). DaT, dopamine transporter; HC, healthy control; PD, Parkinson's disease; SBR, specific binding ratio; SNc, substantia nigra pars compacta.

**Supplementary Table 1. The summary of mediation effects of left SNc iron deposition on striatal DAT-SBR via SNc FPCIT-SBR and diffusion metrics**.

| **Summary** |  |  |  |
| --- | --- | --- | --- |
| X (SNc Iron) | Y (Striatum DaT-SBR) | M1 (SNc FPCIT-SBR) | M2 (SNc-Striatum DTI metrics) |
| SNc-Iron (L) | **Caudate_L** | SNc (L) | **Caudate_L （FA MD AD RD）** |
|  | **aPut_L** |  | **aPut_L （FA MD AD RD）** |
|  | **pPut_L** |  | **pPut_L （FA MD AD RD）** |
|  | **Putamen_L** |  | **Putamen_L （FA MD AD RD）** |
| SNc-Iron (R) | **Caudate_R** | SNc (R) | **Caudate_R （FA MD AD RD）** |
|  | **aPut_R** |  | **aPut_R （FA MD AD RD）** |
|  | **pPut_R** |  | **pPut_R （FA MD AD RD）** |
|  | **Putamen_R** |  | **Putamen_R （FA MD AD RD）** |

**Supplementary Table 2. Mediation effects of SNc iron deposition on bilateral caudate DAT-SBR via SNc FPCIT-SBR and diffusion metrics**.

| SNc-Iron ~ SNc- FPCIT-SBR ~ SNc- Caudate-FA MD AD RD ~ Caudate-DaT-SBR | | | | | | | | | | | | |
| --- | --- | --- | --- | --- | --- | --- | --- | --- | --- | --- | --- | --- |
| **Variable** | | | | **Effect** | | | | | | **Indirect effect** | | |
| **X** | **M1** | **M2** | **Y** | **a1** | **a2** | **b1** | **b2** | **d21** | **c'** | Ind1 | Ind2 | Ind3 |
| SNc-Iron (L) | SNc-DaT (L) | Caudate-FA (L) | Caudate-DaT (L) | -0.283  *p*=0.044 | -0.076  *p*=0.600 | 0.350  *p*=0.005 | 0.021  *p*=0.857 | -0.165  *p*=0.288 | -0.151  *p*=0.178 | **β=-0.099**  **[-0.206, -0.015]** | β=-0.002  [-0.029, 0.070] | β=0.001  [-0.023, 0.020] |
| SNc-Iron (L) | SNc-DaT (L) | Caudate-MD (L) | Caudate-DaT (L) | -0.283  *p*=0.044 | -0.192  *p*=0.190 | 0.350  *p*=0.005 | -0.092  *p*=0.438 | 0.038  *p*=0.805 | -0.171  *p*=0.134 | **β=-0.099**  **[-0.212, -0.016]** | β=0.018  [-0.037, 0.094] | β=0.001  [-0.010, 0.016] |
| SNc-Iron (L) | SNc-DaT (L) | Caudate-AD (L) | Caudate-DaT (L) | -0.283  *p*=0.044 | -0.241  *p*=0.083 | 0.340  *p*=0.006 | -0.065  *p*=0.604 | -0.096  *p*=0.512 | -0.169  *p*=0.146 | **β=-0.096**  **[-0.206, -0.012]** | β=0.016  [-0.055, 0.089] | β=-0.002  [-0.016, 0.008] |
| SNc-Iron (L) | SNc-DaT (L) | Caudate-RD (L) | Caudate-DaT (L) | -0.283  *p*=0.044 | -0.075  *p*=0.611 | 0.355  *p*=0.004 | -0.075  *p*=0.518 | 0.121  *p*=0.445 | -0.159  *p*=0.157 | **β=-0.101**  **[-0.211, -0.014]** | β=0.006  [-0.025, 0.085] | β=0.003  [-0.014, 0.023] |
| SNc-Iron (R) | SNc-DaT (R) | Caudate-FA (R) | Caudate-DaT (R) | -0.366  *p*=0.006 | 0.241  *p*=0.145 | 0.346  *p*=0.004 | -0.025  *p*=0.805 | -0.015  *p*=0.931 | -0.183  *p*=0.096 | **β=-0.127**  **[-0.254, -0.028]** | β=-0.006  [-0.069, 0.041] | β=0.000  [-0.008, 0.021] |
| SNc-Iron (R) | SNc-DaT (R) | Caudate-MD (R) | Caudate-DaT (R) | -0.366  *p*=0.006 | -0.299  *p*=0.051 | 0.348  *p*=0.004 | 0.010  *p*=0.929 | -0.137  *p*=0.404 | -0.186  *p*=0.098 | **β=-0.127**  **[-0.252, -0.029]** | β=-0.003  [-0.080, 0.064] | β=0.000  [-0.009, 0.027] |
| SNc-Iron (R) | SNc-DaT (R) | Caudate-AD (R) | Caudate-DaT (R) | -0.366  *p*=0.006 | -0.206  *p*=0.148 | 0.343  *p*=0.005 | -0.020  *p*=0.860 | -0.172  *p*=0.266 | -0.193  *p*=0.080 | **β=-0.125**  **[-0.250, -0.030]** | β=0.004  [-0.054, 0.056] | β=-0.001  [-0.021, 0.021] |
| SNc-Iron (R) | SNc-DaT (R) | Caudate-RD (R) | Caudate-DaT (R) | -0.366  *p*=0.006 | -0.298  *p*=0.063 | 0.349  *p*=0.004 | 0.018  *p*=0.859 | -0.104  *p*=0.545 | -0.184  *p*=0.101 | **β=-0.127**  **[-0.252, -0.029]** | β=-0.005  [-0.079, 0.055] | β=0.001  [-0.006, 0.026] |

**Note**: Ind1 = a₁ × b₁ (SNc iron → SNc DaT-SBR → striatal DaT-SBR);

Ind2 = a₂ × b₂ (SNc iron → SNc–striatal diffusion metrics → striatal DaT-SBR);

Ind3 = a₁ × d₂₁ × b₂ (serial mediation).

**Supplementary Table 3. Mediation effects of SNc iron deposition on bilateral aPut DAT-SBR via SNc FPCIT-SBR and diffusion metrics**.

| SNc-Iron ~ SNc FPCIT-SBR ~ aPut- FA MD AD RD ~ aPut-DaT-SBR | | | | | | | | | | | | |
| --- | --- | --- | --- | --- | --- | --- | --- | --- | --- | --- | --- | --- |
| **Variable** | | | | **Effect** | | | | | | **Indirect effect** | | |
| **X** | **M1** | **M2** | **Y** | **a1** | **a2** | **b1** | **b2** | **d21** | **c'** | Ind1 | Ind2 | Ind3 |
| SNc-Iron (L) | SNc-DaT (L) | aPut-FA (L) | aPut-DaT (L) | -0.283  *p*=0.044 | -0.080  *p*=0.601 | 0.488  *p*=0.001 | -0.116  *p*=0.353 | -0.131  *p*=0.426 | -0.149  *p*=0.231 | **β=-0.138**  **[-0.265, -0.025]** | β=0.009  [-0.034, 0.088] | β=-0.004  [-0.030, 0.011] |
| SNc-Iron (L) | SNc-DaT (L) | aPut-MD (L) | aPut-DaT (L) | -0.283  *p*=0.044 | -0.075  *p*=0.625 | 0.504  *p*<0.0001 | 0.012  *p*=0.924 | -0.134  *p*=0.414 | -0.138  *p*=0.268 | **β=-0.143**  **[-0.276, -0.026]** | β=-0.001  [-0.103, 0.033] | β=0.000  [-0.014, 0.020] |
| SNc-Iron (L) | SNc-DaT (L) | aPut-AD (L) | aPut-DaT (L) | -0.283  *p*=0.044 | -0.182  *p*=0.216 | 0.485  *p*=0.001 | -0.070  *p*=0.596 | -0.252  *p*=0.109 | -0.152  *p*=0.230 | **β=-0.137**  **[-0.268, -0.024]** | β=0.013  [-0.086, 0.080] | β=-0.005  [-0.031, 0.021] |
| SNc-Iron (L) | SNc-DaT (L) | aPut-RD (L) | aPut-DaT (L) | -0.283  *p*=0.044 | -0.003  *p*=0.982 | 0.505  *p*<0.0001 | 0.048  *p*=0.699 | -0.044  *p*=0.789 | -0.139  *p*=0.264 | **β=-0.143**  **[-0.271, -0.031]** | β=0.000  [-0.083, 0.039] | β=0.001  [-0.092, 0.043] |
| SNc-Iron (R) | SNc-DaT (R) | aPut-FA (R) | aPut-DaT (R) | -0.366  *p*=0.006 | 0.272  *p*=0.098 | 0.584  *p*<0.0001 | -0.050  *p*=0.651 | 0.018  *p*=0.920 | -0.102  *p*=0.391 | **β=-0.213**  **[-0.363, -0.072]** | β=-0.013  [-0.081, 0.039] | β=0.000  [-0.011, 0.014] |
| SNc-Iron (R) | SNc-DaT (R) | aPut-MD (R) | aPut-DaT (R) | -0.366  *p*=0.006 | -0.364  *p*=0.015 | 0.592  *p*<0.0001 | 0.047  *p*=0.700 | -0.191  *p*=0.230 | -0.099  *p*=0.426 | **β=-0.216**  **[-0.369, -0.072]** | β=-0.017  [-0.091, 0.055] | β=0.003  [-0.014, 0.025] |
| SNc-Iron (R) | SNc-DaT (R) | aPut-AD (R) | aPut-DaT (R) | -0.366  *p*=0.006 | -0.253  *p*=0.085 | 0.590  *p*<0.0001 | 0.032  *p*=0.794 | -0.206  *p*=0.194 | -0.108  *p*=0.369 | **β=-0.216**  **[-0.371, -0.069]** | β=-0.008  [-0.063, 0.043] | β=0.002  [-0.017, 0.025] |
| SNc-Iron (R) | SNc-DaT (R) | aPut-RD (R) | aPut-DaT (R) | -0.366  *p*=0.006 | -0.353  *p*=0.024 | 0.589  *p*<0.0001 | 0.042  *p*=0.719 | -0.149  *p*=0.0.367 | -0.101  *p*=0.410 | **β=-0.215**  **[-0.384, -0.072]** | β=-0.015  [-0.089, 0.052] | β=0.002  [-0.012, 0.022] |

**Note**: Ind1 = a₁ × b₁ (SNc iron → SNc DaT-SBR → striatal DaT-SBR);

Ind2 = a₂ × b₂ (SNc iron → SNc–striatal diffusion metrics → striatal DaT-SBR);

Ind3 = a₁ × d₂₁ × b₂ (serial mediation).

**Supplementary Table 4. Mediation effects of SNc iron deposition on bilateral pPut DAT-SBR via SNc FPCIT-SBR and diffusion metrics**.

| SNc-Iron ~ SNc FPCIT-SBR ~ pPut- FA MD AD RD ~ pPut-DaT-SBR | | | | | | | | | | | | |
| --- | --- | --- | --- | --- | --- | --- | --- | --- | --- | --- | --- | --- |
| **Variable** | | | | **Effect** | | | | | | **Indirect effect** | | |
| **X** | **M1** | **M2** | **Y** | **a1** | **a2** | **b1** | **b2** | **d21** | **c'** | Ind1 | Ind2 | Ind3 |
| SNc-Iron (L) | SNc-DaT (L) | pPut-FA (L) | pPut-DaT (L) | -0.283  *p*=0.044 | -0.102  *p*=0.506 | 0.466  *p*<0.0001 | -0.226  *p*=0.052 | -0.069  *p*=0.672 | -0.162  *p*=0.154 | **β=-0.132**  **[-0.253, -0.028]** | β=0.023  [-0.056, 0.114] | β=-0.004  [-0.029, 0.022] |
| SNc-Iron (L) | SNc-DaT (L) | pPut-MD (L) | pPut-DaT (L) | -0.283  *p*=0.044 | 0.021  *p*=0.893 | 0.481  *p*<0.0001 | 0.136  *p*=0.230 | 0.011  *p*=0.950 | -0.142  *p*=0.221 | **β=-0.136**  **[-0.257, -0.032]** | β=0.003  [-0.101, 0.063] | β=0.000  [-0.017, 0.016] |
| SNc-Iron (L) | SNc-DaT (L) | pPut-AD (L) | pPut-DaT (L) | -0.283  *p*=0.044 | -0.122  *p*=0.434 | 0.481  *p*<0.0001 | -0.101  *p*=0.386 | -0.013  *p*=0.935 | -0.152  *p*=0.199 | **β=-0.136**  **[-0.262, -0.027]** | β=0.012  [-0.053, 0.116] | β=0.000  [-0.015, 0.022] |
| SNc-Iron (L) | SNc-DaT (L) | pPut-RD (L) | pPut-DaT (L) | -0.283  *p*=0.044 | 0.075  *p*=0.632 | 0.479  *p*<0.0001 | 0.197  *p*=0.083 | 0.017  *p*=0.917 | -0.154  *p*=0.178 | **β=-0.136**  **[-0.251, -0.029]** | β=0.015  [-0.086, 0.098] | β=-0.001  [-0.022, 0.020] |
| SNc-Iron (R) | SNc-DaT (R) | pPut-FA (R) | pPut-DaT (R) | -0.371  *p*=0.006 | 0.136  *p*=0.400 | 0.571  *p*<0.0001 | -0.167  *p*=0.102 | -0.054  *p*=0.757 | -0.097  *p*=0.357 | **β=-0.212**  **[-0.353, -0.079]** | β=-0.023  [-0.122, 0.023] | β=-0.003  [-0.025, 0.023] |
| SNc-Iron (R) | SNc-DaT (R) | pPut-MD (R) | pPut-DaT (R) | -0.371  *p*=0.006 | -0.218  *p*=0.171 | 0.604  *p*<0.0001 | 0.129  *p*=0.220 | -0.187  *p*=0.279 | -0.092  *p*=0.397 | **β=-0.224**  **[-0.375, -0.081]** | β=-0.028  [-0.091, 0.019] | β=0.009  [-0.011, 0.038] |
| SNc-Iron (R) | SNc-DaT (R) | pPut-AD (R) | pPut-DaT (R) | -0.371  *p*=0.006 | -0.148  *p*=0.377 | 0.586  *p*<0.0001 | 0.026  *p*=0.794 | -0.256  *p*=0.161 | -0.116  *p*=0.288 | **β=-0.217**  **[-0.369, -0.076]** | β=-0.004  [-0.070, 0.030] | β=0.002  [-0.018, 0.027] |
| SNc-Iron (R) | SNc-DaT (R) | pPut-RD (R) | pPut-DaT (R) | -0.371  *p*=0.006 | -0.203  *p*=0.201 | 0.596  *p*<0.0001 | 0.148  *p*=0.158 | -0.112  *p*=0.512 | -0.090  *p*=0.403 | **β=-0.221**  **[-0.361, -0.080]** | β=-0.030  [-0.105, 0.016] | β=0.006  [-0.017, 0.036] |

**Note**: Ind1 = a₁ × b₁ (SNc iron → SNc DaT-SBR → striatal DaT-SBR);

Ind2 = a₂ × b₂ (SNc iron → SNc–striatal diffusion metrics → striatal DaT-SBR);

Ind3 = a₁ × d₂₁ × b₂ (serial mediation).

**Supplementary Table 5. Mediation effects of SNc iron deposition on bilateral putamen DAT-SBR via SNc FPCIT-SBR and diffusion metrics**.

| SNc-Iron ~ SNc FPCIT-SBR ~ Putamen- FA MD AD RD ~ Putamen-DaT-SBR | | | | | | | | | | | | |
| --- | --- | --- | --- | --- | --- | --- | --- | --- | --- | --- | --- | --- |
| **Variable** | | | | **Effect** | | | | | | **Indirect effect** | | |
| **X** | **M1** | **M2** | **Y** | **a1** | **a2** | **b1** | **b2** | **d21** | **c'** | Ind1 | Ind2 | Ind3 |
| SNc-Iron (L) | SNc-DaT (L) | Putamen -FA (L) | Putamen -DaT (L) | -0.283  *p*=0.044 | -0.101  *p*=0.517 | 0.476  *p*<0.0001 | -0.187  *p*=0.112 | -0.123  *p*=0.459 | -0.161  *p*=0.172 | β=-0.135  [-0.261, -0.024] | β=0.019  [-0.044, 0.107] | β=-0.006  [-0.032, 0.018] |
| SNc-Iron (L) | SNc-DaT (L) | Putamen -MD (L) | Putamen -DaT (L) | -0.283  *p*=0.044 | -0.044  *p*=0.783 | 0.503  *p*<0.0001 | 0.082  *p*=0.479 | -0.047  *p*=0.784 | -0.138  *p*=0.248 | β=-0.142  [-0.273, -0.027] | β=-0.004  [-0.099, 0.032] | β=0.001  [-0.008, 0.017] |
| SNc-Iron (L) | SNc-DaT (L) | Putamen -AD (L) | Putamen -DaT (L) | -0.283  *p*=0.044 | -0.183  *p*=0.227 | 0.484  *p*<0.0001 | -0.103  *p*=0.403 | -0.141  *p*=0.381 | -0.161  *p*=0.187 | β=-0.137  [-0.264, -0.024] | β=0.019  [-0.061, 0.118] | β=-0.004  [-0.023, 0.017] |
| SNc-Iron (L) | SNc-DaT (L) | Putamen -RD (L) | Putamen -DaT (L) | -0.283  *p*=0.044 | 0.033  *p*=0.839 | 0.498  *p*<0.0001 | 0.139  *p*=0.225 | 0.010  *p*=0.955 | -0.146  *p*=0.216 | β=-0.141  [-0.267, -0.029] | β=0.005  [-0.085, 0.063] | β=0.000  [-0.014, 0.019] |
| SNc-Iron (R) | SNc-DaT (R) | Putamen-FA (R) | Putamen-DaT (R) | -0.372  *p*=0.007 | 0.195  *p*=0.245 | 0.589  *p*<0.0001 | -0.106  *p*=0.314 | -0.046  *p*=0.797 | -0.095  *p*=0.399 | β=-0.219  [-0.371, -0.076] | β=-0.021  [-0.094, 0.020] | β=-0.002  [-0.017, 0.017] |
| SNc-Iron (R) | SNc-DaT (R) | Putamen-MD (R) | Putamen-DaT (R) | -0.372  *p*=0.007 | -0.258  *p*=0.110 | 0.606  *p*<0.0001 | 0.065  *p*=0.557 | -0.183  *p*=0.293 | -0.099  *p*=0.392 | β=-0.225  [-0.383, -0.076] | β=-0.017  [-0.075, 0.026] | β=0.004  [-0.011, 0.027] |
| SNc-Iron (R) | SNc-DaT (R) | Putamen-AD (R) | Putamen-DaT (R) | -0.372  *p*=0.007 | -0.142  *p*=0.393 | 0.594  *p*<0.0001 | 0.002  *p*=0.988 | -0.240  *p*=0.186 | -0.115  *p*=0.309 | β=-0.221  [-0.376, -0.076] | β=0.000  [-0.053, 0.038] | β=0.000  [-0.020, 0.023] |
| SNc-Iron (R) | SNc-DaT (R) | Putamen-RD (R) | Putamen-DaT (R) | -0.372  *p*=0.007 | -0.259  *p*=0.112 | 0.603  *p*<0.0001 | 0.079  *p*=0.468 | -0.118  *p*=0.499 | -0.095  *p*=0.408 | β=-0.224  [-0.375, -0.076] | β=-0.020  [-0.083, 0.026] | β=0.003  [-0.011, 0.026] |

**Note**: Ind1 = a₁ × b₁ (SNc iron → SNc DaT-SBR → striatal DaT-SBR);

Ind2 = a₂ × b₂ (SNc iron → SNc–striatal diffusion metrics → striatal DaT-SBR);

Ind3 = a₁ × d₂₁ × b₂ (serial mediation).

**Supplementary Table 6. Summary of all sequence parameters for the uPMR790 3 T scanner.**

| Sequence | Field of field (mm^2^) | TR (ms) | TE (ms) | Thickness (mm) | matrix | slice |
| --- | --- | --- | --- | --- | --- | --- |
| PET | 300x300 | NA | NA | 2 | 192x192 | 160 |
| T1 | 256x256 | 7.9 | 2.8 | 1 | 256x256 | 176 |
| QSM | 256x256 | 28.3 | 3.4 | 1 | 256x256 | 118 |
| DTI | 230x240 | 8632 | 84.9 | 2.5 | 256x256 | 60 |

**Supplementary Table 7. Demographic of one PD and one control obtained by the Netherlands Brain Bank.**

| **demographic** | **PD** | **control** |
| --- | --- | --- |
| PMD | 04:55 | 07:30 |
| Lewy body stage | 6 | 0 |
| Braak Stage | 1 | 1 |
| CERAD | O | O |
| sex | male | female |
| Age (years) | 67 | 55 |

**Supplementary Table 8. List of primary antibodies used for western blotting and immunofluorescence staining.**

| **Antibody/Compounds** | **Company** | **Cat. No.** | **Dilution** |
| --- | --- | --- | --- |
| 6E10, Anti-β-amyloid, 1-16 antibody - Mouse | Biolegend | 803001 | 1:1000 |
| AT-8, anti-phospho-tau Ser202, Thr205 - Mouse | Invitrogen | MN1020 | 1:1000 |
| Anti-Alpha-synuclein pS129 (phospho-Ser129) – Rabbit [EP1536Y] | Abcam | ab51253 | 1:1000 |
| Dopamine transporter | Merck Millipore | MAB369 | 1:1000 |
| Alexa Fluor® 555 Anti-Alpha-synuclein (phospho S129) antibody [EP1536Y] | Abcam | ab313137 | 1:1000 |
| Tyrosine Hydroxylase Antibody | Merck Millipore | AB152 | 1:1000 |
| MBP Polyclonal Antibody- Rabbit | ThermoFisher | PA5-78397 | 1:1000 |
| DAPI | Sigma‒Aldrich | D9542-10MG | 1:1000 |
| VECTASHIELD® Antifade | Vector Laboratories | H-1000-10 |  |
| Normal goat Serum (NGS) | Chemie Brunschwig | JAC005-000-121 |  |
| Normal Donkey Serum (NDS) | Interchim | UP77719A K |  |
| Fomblin Y, LVAC 16/6, average molecular weight 2700 | Merck | 317942 |  |
| Reagents Ethanol 95%, 2% MEK | Biosystems Switzerland AG | 84-3205-00-PE |  |
| Luxol Fast Blue | Biosystems Switzerland AG | 11125.00500 |  |
| Lithiumcarbonat 0.05% | Biosystems Switzerland AG | 11714.00500 |  |
| Essigsäure / Eisessig 100% zur Analyse | Biosystems Switzerland AG | M100063.1000 |  |
| Kresylviolett 0,25% wässrig | Biosystems Switzerland AG | 11765.00500 |  |
| Aqua dem. Ethanol 70%, 2% MEK | Biosystems Switzerland AG | 84-3305-00-PE |  |
| Isopropanol 100%, min. 99,7% | Biosystems Switzerland AG | 84-4205-00-PE |  |
| Xylol Isomerengemisch | Biosystems Switzerland AG | 84-2505-00-PE |  |
| CV Ultra Eindeckmittel | Biosystems Switzerland AG | 14070937891 |  |

**Supplementary Table 9. Abbreviations list**

| **Abbreviation** | **Full term** | **Abbreviation** | **Full term** |
| --- | --- | --- | --- |
| PD | Parkinson’s disease | L | left hemisphere |
| SNc | substantia nigra pars compacta | R | right hemisphere |
| DaT | dopamine transporter | H&E | Hematoxylin & Eosin |
| PET | positron emission tomography | ppb | parts per billion |
| pPut | posterior putamen | pS129 | phospho-α-synuclein |
| QSM | quantitative susceptibility mapping | DAPI | 4’, 6-diamidino-2-phenylindole |
| DTI | diffusion tensor imaging | FW | free water |
| AD | axial diffusivity | MBP | myelin basic protein |
| RD | radial diffusivity | Cd | caudate |
| MD | mean diffusivity | TH | tyrosine hydroxylase |
| FA | fractional anisotropy | FWHM | full width at half maximum |
| ROC | receiver operating characteristic | FOV | field of view |
| AUC | area under the curve | TR | repetition time |
| PFF | preformed fibril | TE | echo time |
| HCs | healthy controls | GRE | Gradient-Recalled Echo |
| UPDRS III | Unified Parkinson's Disease Rating Scale Part III | PVC | partial-volume correction |
| MMSE | Mini-Mental State Examination | FOD | fiber orientation distributions |
| SWI | susceptibility weighted imaging | NTLs | non-transgenic littermates |
| CIs | confidence intervals |  |  |
